# Amyloid‐Templated Ceria Nanozyme Reinforced Microneedle for Diabetic Wound Treatments

**DOI:** 10.1002/adma.202417774

**Published:** 2025-02-25

**Authors:** Qize Xuan, Jiazhe Cai, Yuan Gao, Xinchi Qiao, Tonghui Jin, Mohammad Peydayesh, Jiangtao Zhou, Qiyao Sun, Lijian Zhan, Bin Liu, Ping Wang, Hui Li, Chao Chen, Raffaele Mezzenga

**Affiliations:** ^1^ Institute for Environmental Pollution and Health, School of Environmental and Chemical Engineering Shanghai University Shanghai 200444 P. R. China; ^2^ Department of Health Sciences and Technology ETH Zürich Schmelzbergstrasse 9 Zürich 8092 Switzerland; ^3^ State Key Laboratory of Bioreactor Engineering Center, School of Biotechnology East China University of Science and Technology Shanghai 200237 China; ^4^ Institute for Biomedical Engineering ETH Zürich Zürich 8092 Switzerland; ^5^ Department of Bioproducts and Biosystems Engineering University of Minnesota St Paul MN 55108 USA; ^6^ Department of Materials ETH Zürich Wolfgang‐Pauli‐Strasse 10 Zürich 8049 Switzerland

**Keywords:** amyloid fibrils, ceria nanozymes, diabetic wound, microenvironment regulation, microneedle

## Abstract

Amyloid fibrils have emerged as excellent templates and building blocks for the development of ordered functional materials with considerable potential in biomedical applications. Here, lysozyme amyloid fibrils (Lys‐AFs) are employed as templates for the in situ synthesis of ceria nanozymes (Lys‐AFs‐Ceria) with ultrafine dimensions, an optimized Ce^3+^/Ce^4+^ ratio, and uniform distribution on the fibril surface, addressing the challenges of low catalytic efficiency and high susceptibility to aggregation typical of traditional methods. As a proof of concept, it is further applied Lys‐AFs‐Ceria to develop hydrogel/microneedle for treating bacteria‐infected diabetic wounds via non‐covalent interactions between polyphenols and amyloid fibrils incorporating glucose oxidase (GOX). The hydrogel/microneedle facilitates superoxide dismutase and catalase cascade catalysis by Lys‐AFs‐Ceria, and integrates GOX‐mediated glucose consumption, synergistically achieving glucose reduction, reactive oxygen species elimination, and hypoxia alleviation in the diabetic wound infection microenvironment. In addition to antibacterial properties and tissue regeneration promotion of Lys‐AFs scaffold, Lys‐AFs‐Ceria regulates macrophages polarization toward an anti‐inflammatory M2 state. Collectively, these attributes contribute to the enhanced efficacy of diabetic wound healing, with in vivo studies demonstrating increased healing efficiency following a single application, and more in general an effective strategy toward high‐catalytic and stable nanozymes.

## Graphical abstract

This work demonstrates an effective strategy for synthesizing high‐catalytic and stable nanozymes, and introduces the first example of amyloid‐ceria nanozymes reinforced microneedle which features a complex, synergistic catalytic behavior combining superoxide dismutase and catalase‐like activities, promoting to remodel the infection microenvironment in diabetic wounds, and holding significant promise for clinical applications.

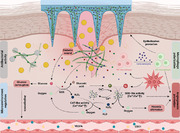

## 1 Introduction

The pathological significance of amyloidosis, characterized by amyloidogenic protein aggregation and amyloid deposition, has long been recognized in the context of neurological disorders.^[^ ^1^ ^]^ Nevertheless, recent breakthroughs have illuminated the functional roles of amyloid fibrils in microorganisms.^[^ ^2^ ^]^ These discoveries expand our understanding, and also drive advancements in the design of proteinaceous materials based on the amyloid fibrils.^[^ ^3^ ^]^ Artificial amyloids, generated through the controlled self‐assembly of protein or polypeptides, exhibit the hallmark of repeated cross‐β sheet structures, orienting perpendicular to the fibril axis and interconnecting with adjacent sheets.^[^ ^4^ ^]^ The variability in fibril morphologies, mechanical properties and surface functionalities depends upon the protein sequence,^[^ ^4b^ ^]^ suggesting that precise sequence programming can direct amyloid fabrication to achieve desired features.^[^ ^3a^ ^]^

These distinctive structures endow amyloid fibrils a rich array of physicochemical properties, particularly in structural stability and mechanical resilience. The β‐sheet structure in the amyloid core exhibits remarkable robustness, with some amyloid fibrils exhibiting mechanical strengths comparable to spider silk, in some cases, even rivaling that of steel.^[^ ^5^ ^]^ Notably, these structural and mechanical attributes are remarkably conservative, independent of the specific protein sequence or chemical modifications.^[^ ^5b^ ^]^ This extraordinary structural integrity allows amyloid fibrils to endure a range of extreme conditions, including temperatures variation, proteolytic degradation, surfactants, and substantial mechanical forces,^[^ ^6^ ^]^ enabling them ideal scaffolds for diverse applications. Furthermore, the sequence‐related surface chemistry of amyloid fibrils confers additional material functionality.^[^ ^7^ ^]^ Consequently, amyloid fibrils can serve as versatile templates,^[^ ^8^ ^]^ exemplified by their ability to facilitate the binding of metal ions and the synthesis of inorganic nanoparticles via supramolecular metal–ligand interactions,^[^ ^9^ ^]^ such as amyloid fibrils templates for the production of gold crystals^[^ ^10^ ^]^ and iron nanoparticles.^[^ ^11^ ^]^ The bioactivities of amyloid fibrils further enhance their utility, making them promising candidates for diverse biomedical applications.^[^ ^12^ ^]^ For example, amyloid fibrils exhibit generic cell adhesion properties, even in the absence of specific integrin recognition motifs.^[^ ^13^ ^]^ They actively promote cell adhesion and spreading by recruiting integrin and facilitating the formation of focal adhesion complexes, mimicking the behavior of extracellular matrix (ECM) proteins.^[^ ^12^, ^13^, ^14^ ^]^ Additionally, the antibacterial activity of amyloid fibrils has been highlighted due to their exposed positively charged residues,^[^ ^15^ ^]^ with enhanced performance over certain antibacterial peptides.^[^ ^16^ ^]^ Taken together, the diverse physicochemical properties and bioactivities including the biocompatibility and ECM mimicry, and antibacterial activity position amyloid fibrils as exceptional biomaterial templates and bioactive building blocks for constructing ordered nanomaterials with considerable potential in biomedical applications, including in the context of tissue repair.

Nanozymes, denoting nanomaterials with intrinsic enzyme‐like capabilities, represent a promising avenue for non‐invasive therapy^[^ ^17^ ^]^ and disease treatments,^[^ ^18^ ^]^ with a specific focus on their potential in managing diabetic wounds.^[^ ^19^ ^]^ Diabetic wounds are notoriously difficult to treat due to complex factors, including persistent bacterial infections, elevated oxidative stress and glucose concentration, impaired angiogenesis, and hyperinflammation, often leading to non‐healing ulcers or even amputations.^[^ ^20^ ^]^ However, present treatments typically address only one aspect of the problem (e.g., controlling infection or reducing inflammation) without providing a comprehensive solution, especially for infection microenvironment improvements.^[^ ^21^ ^]^ Diabetic wounds are typically marked by increasing levels of reactive oxygen species and hypoxia, particularly superoxide anions (O_2_^•−^) and hydrogen peroxide (H_2_O_2_), which severely impaired the angiogenesis and healing.^[^ ^22^ ^]^ Nanozymes, such as ceria,^[^ ^23^ ^]^ Prussian blue,^[^ ^24^ ^]^ manganese oxide,^[^ ^25^ ^]^ and metal‐organic frameworks,^[^ ^26^ ^]^ offer distinct advantages within diabetic wounds due to their capacity to catalyze and regulate the adverse microenvironments. Ceria nanozymes, in particular, have attracted increasing attention due to their multiple enzymatic‐like activities. However, one of the main challenges remains in optimizing the Ce^3+^/Ce^4+^ ratio, because a higher ratio promotes superoxide dismutase (SOD)‐like activity while a lower ratio facilitates catalase (CAT)‐like activity.^[^ ^27^ ^]^ Further, synthesizing ultrafine nanosized ceria nanozymes (less than 10 nm) while enhancing catalytic activity and preventing aggregation poses substantial challenges. In addition, traditional synthesis process still relies strictly on harsh reaction conditions and the use of organic solvents. This calls for an urgent need to develop innovative synthetic strategies and enhance both the catalytic activity and long‐term stability of ceria nanozymes.

**Figure 1:**
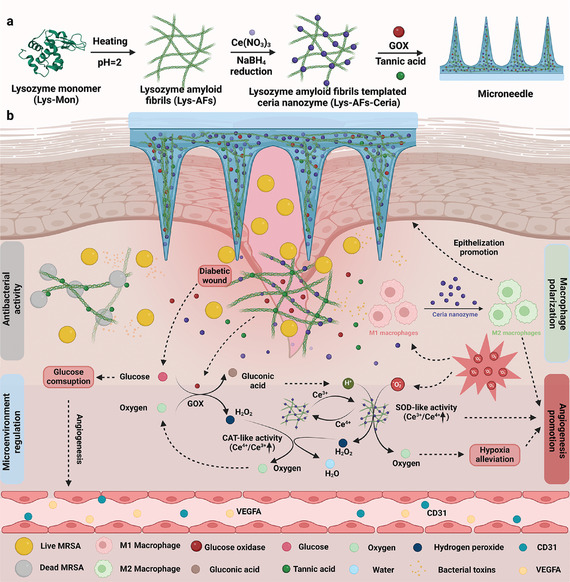
Amyloid‐templated ceria nanozyme reinforced microneedle for diabetic wounds treatment. a) Schematic representation of the fabrication process of amyloid‐templated ceria nanozyme reinforced microneedle. b) Microneedle promotes the healing of diabetic wounds via a combination of synergistic mechanisms, including microenvironment regulation, antibacterial activity, macrophage polarization, and angiogenesis promotion. This Figure was created with www.biorender.com.

Concerning the versatile roles of amyloid fibrils, herein, we have utilized lysozyme amyloid fibrils (Lys‐AFs) as templates for the in situ synthesis of ceria nanozymes (Lys‐AFs‐Ceria) to address challenges related to catalytic efficiency and aggregation issues from traditional nanozyme synthesis methods. Leveraging the outstanding properties of amyloid fibrils in terms of mechanical strengths, surface chemistry, and bioactivities, this innovative template strategy is expected to yield ceria nanozymes with smaller sizes, improved dispersity, and reduced aggregation tendencies. This, in turn, significantly enhances their catalytic activity and long‐term stability. To further demonstrate the application potential of this strategy, we engineered hydrogel microneedles using tannic acid as an inducer and incorporated glucose oxidase (GOX) for treating bacterial‐infected diabetic wounds. As illustrated in **Figure** **1**, the prepared microneedles are designed to promote the healing of diabetic wounds through several mechanisms. Initially, GOX catalyzes the conversion of high glucose concentrations into gluconic acid and hydrogen peroxide (H_2_O_2_). Simultaneously, Lys‐AFs‐Ceria primarily exhibits SOD‐like activity due to a higher Ce^3+^/Ce^4+^ ratio, efficiently converting O_2_^•−^into H_2_O_2_ and oxygen. As the Ce^3+^/Ce^4+^ ratio decreases, Lys‐AFs‐Ceria then predominantly displays CAT‐like activity, where it effectively converts all generated H_2_O_2_ into oxygen and H_2_O. This dual enzymatic activity regulates the local microenvironment, mitigate oxidative stress, and promote angiogenesis, significantly contributing to the effective healing of diabetic wounds. Moreover, the lysozyme amyloid fibrils themselves possess remarkable inherent broad‐spectrum antimicrobial properties and serve as ECM components, enhancing cell adhesion, spreading, and proliferation in diabetic wounds. Additionally, Lys‐AFs‐Ceria shifts macrophage polarization from pro‐inflammatory M1 phenotype to the anti‐inflammatory M2 phenotype, further mitigating excessive inflammation and promoting epithelial regeneration at wound sites. In brief, our work provides a comprehensive regulation strategy for infected diabetic wound microenvironment, in which amyloid fibrils act as synthesis templates and bioactive scaffolds, and enhance the catalytic stability of nanozymes. This work also offers an efficient and stable strategy for nanozyme synthesis, and presents a clinically promising material for the treatment of diabetic wounds.

## 2 Results and Discussion

### 2.1 Amyloid Fibrils‐Templated in situ Synthesis of Ceria Nanozyme (Lys‐AFs‐Ceria)

**Figure 2:**
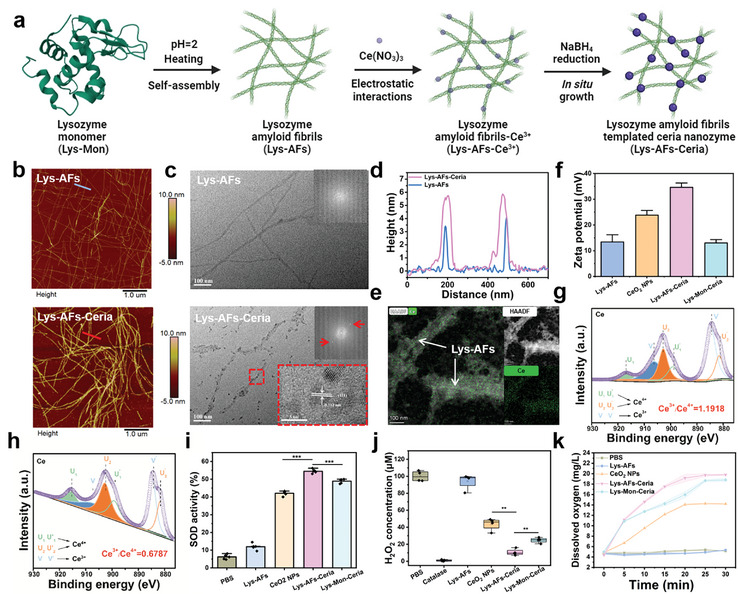
Characterization of amyloid fibrils‐templated in situ synthesis of ceria nanozymes and their catalytic properties. a) The schematic procedure of amyloid‐templated ceria nanozyme synthesis. This panel was created with www.biorender.com. b) AFM images and c) TEM images of lysozyme amyloid fibrils (Lys‐AFs) and lysozyme amyloid fibrils‐ceria nanozyme (Lys‐AFs‐Ceria). d) Height profile of Lys‐AFs and Lys‐AFs‐Ceria from panel b. e) Element mapping analysis of Lys‐AFs‐Ceria. f) Equivalent zeta potential results (*n* = 3) of different groups including Lys‐AFs, CeO_2_ NPs, Lys‐AFs‐Ceria, and Lys‐Mon‐Ceria (lysozyme monomer‐templated ceria nanozyme). High‐resolution Ce 3d spectra of g) Lys‐AFs‐Ceria and h) CeO_2_ NPs. The C 1s binding energy value (284.6 eV) was used as a reference to calibrate the binding energy values of other elements. i) SOD‐like activity assays (*n* = 5) of different samples. j) Residual H_2_O_2_ concentration assays (*n* = 4) of different samples. k) Dissolved oxygen assays (*n* = 3) of H_2_O_2_ solution after different treatments (PBS, Lys‐AFs, CeO_2_ NPs, Lys‐AFs‐Ceria, and Lys‐Mon‐Ceria). Statistical significance was defined as *p < 0.05, **p < 0.01 and ***p < 0.001.

In this work, Lys‐AFs were employed as templates for the synthesis of ceria nanozymes (**Figure** **2**a). Lysozyme derived from hen egg‐white was selected to prepare amyloid fibrils owing to its widespread availability, excellent dispersibility, antibacterial properties and abundant functional groups. The functional group on the surface of Lys‐AFs promoted the homogenous nanosized ceria nanozyme distribution on the fibrils, to ensure the enhanced catalytic activity. Lys‐AFs with a height of ≈3 nm demonstrated the excellent mechanical rigidity^[^ ^28^ ^]^ after heating lysozyme monomer solution (pH 2) at 90 °C for 24 h (Figure 2b). The ceria ions were then introduced to bind specific residues of Lys‐AFs surface and a reduction process mediated by sodium borohydride (NaBH_4_) was performed to prepare ceria nanozymes on the surface of Lys‐AFs (Lys‐AFs‐Ceria), resulting in an increased fibrils height (Figure 2b,d). These ceria nanoparticles have a diameter of less than 5 nm on the surface of Lys‐AFs, and a lattice spacing measured at 0.312 nm (Figure 2c,e), showing the successful synthesis of nanoceria with ultrafine properties and uniform distribution.^[^ ^29^ ^]^ Nanopore testing results, as exhibited in Figure S1 (Supporting Information), indicated changes in current (*△I*) were directly proportional to the diameter of amyloid fibrils through the nanopores, with a 42 pA difference between Lys‐AFs and Lys‐AFs‐Ceria groups, confirming that nanoceria modification on the surface increased the diameter of Lys‐AFs. In contrast, both the diameter of pure nanoceria (CeO_2_ NPs) and lysozyme monomer‐templated nanoceria (Lys‐Mon‐Ceria) exceeded 10 nm (Figures S2 and S3, Supporting Information), obviously larger than Lys‐AFs‐Ceria which was only ≈3 nm. These findings unequivocally demonstrated the potential of Lys‐AFs to act as a template in synthesizing ultrafine and homogenously‐dispersed ceria nanozymes.

To further investigate the underlying binding mechanism of ceria, electrophoretic mobility assays were conducted. Both Lys‐AFs and CeO_2_ NPs group displayed positive charges, corresponding to an equivalent zeta potential of 13.40±2.79 and 23.83±1.81 mV, respectively. Although Lys‐AFs exhibited an overall positive charge at pH 7, they retained numerous sites with negatively charged amino acids, such as aspartic acid and glutamic acid.^[^ ^30^ ^]^ These sites provide feasible attachment points for ceria ions and further facilitate the in situ growth of nanoceria. Consequently, the introduction of nanoceria significantly increased the positive charge to 34.63±1.66 mV for Lys‐AFs‐Ceria (Figure 2f). This increased positive charge suggests that Lys‐AFs‐Ceria will have a more robust binding ability with bacteria. Additionally, Lys‐Mon‐Ceria exhibited an equivalent zeta potential similar with that of Lys‐AFs, attributed to the presence of surface‐bound unreacted lysozyme monomers (Figures S2 and S3, Supporting Information). These amorphous structures were evidenced by the broad peak of 20–25 degree in XRD data (Figure S4, Supporting Information). In contrast, Lys‐AFs‐Ceria exhibited the similar curve with CeO_2_ NPs, confirming the binding of nanoceria on the Lys‐AFs surface. To accurately quantify the ratio of binding Ce ions, the reaction solution after NaBH_4_ reduction was centrifuged and the supernatant was taken out to determine the unbinding Ce ions content through ICP‐MS assays. A shown in Figure S5 (Supporting Information), the original concentration of Ce ions before NaBH_4_ reduction was 58.43 mg L^−1^, while the Ce concentrations in supernatant was 36.3 mg L^−1^, and thus the ratio of Ce ions binding is ≈37.87%.

The surface chemical composition of Lys‐AFs‐Ceria and the valence state of nanoceria, which critically affect their catalytic activity,^[^ ^31^ ^]^ were evaluated using X‐ray photoelectron spectroscopy (XPS). Notably, the Ce^3+^/Ce^4+^ ratio of Lys‐AFs‐Ceria was substantially higher than that of CeO_2_ NPs, as demonstrated by high‐resolution XPS data (Figure 2g,h). The presence of Ce^3+^ is essential for the SOD‐like activity, and the high Ce^3+^/Ce^4+^ ratio was largely attributed to the synergistic effect of the reduction process mediated by NaBH_4_ and the inherent reduction capacity of Lys‐AFs.^[^ ^11^ ^]^ We also conducted XPS analysis of Lys‐AFs‐Ceria under varying NaBH_4_ concentrations to examine its effects on the Ce^3^⁺/Ce⁴⁺ ratio. As shown in Figure S6 (Supporting Information), the observed Ce^3^⁺/Ce⁴⁺ ratio at 0.026 M NaBH_4_ likely represents an optimal balance of Ce^3^⁺ concentration and surface oxygen vacancies, maintaining a high redox‐active Ce^3^⁺ fraction, the structural stability, and a lower utilization of reductants.^[^ ^32^ ^]^ The formation of Ce‐O‐C bonds between nanoceria and Lys‐AFs^[^ ^33^ ^]^ was confirmed by the UV‐Vis spectrum (Figure S7, Supporting Information). Additionally, the nanoceria content showed negligible variation across all groups (Figure S8, Supporting Information), thus fulfilling a standard prerequisite for enzyme activity assays.

### 2.2 Catalytic Activity Characterization of Lys‐AFs‐Ceria

The SOD‐like and CAT‐like activities are pivotal characteristics of ceria nanozymes, with their performances predominantly dependent on the Ce^3+^/Ce^4+^ ratio.^[^ ^27^ ^]^ To further demonstrate the advantages of the Lys‐AFs template strategy in enhancing catalytic performance, the SOD‐like activity of CeO_2_ NPs and Lys‐AFs‐Ceria was initially assessed using the standard WST‐1 colorimetric method, in which O_2_^•−^ was produced by xanthine and xanthine oxidase (XOD), reducing WST‐1 to water‐soluble formazan dye with a characteristic peak at 450 nm (Figure S9, Supporting Information). As shown in Figure 2i, as‐prepared Lys‐AFs exhibited a low SOD‐like activity due to reducing capacity of proteinaceous amyloid fibrils.^[^ ^11^ ^]^ Notably, the Lys‐AFs‐Ceria group showed ≈12% higher SOD‐like activity compared to the CeO_2_ NPs group and ≈8% improvement over the Lys‐Mon‐Ceria group. These enhancements in SOD‐like activity of Lys‐AFs‐Ceria could be partly attributed to higher Ce^3+^/Ce^4+^ ratio as discussed earlier (Figure 2g,h), and in part to homogenous nanosized ceria nanozyme (Figure 2b,d). Another significant challenge for ceria nanozymes is their tendency to aggregate, leading to a dramatic reduction of activity. To evaluate their stability, all samples encompassing CeO_2_ NPs, Lys‐AFs‐Ceria, and Lys‐Mon‐Ceria, were kept at room temperature for the duration of one month. A comparison was made between these stored samples and their corresponding freshly‐prepared counterparts in terms of their dispersion properties and SOD‐like activity. As shown in Figure S10 (Supporting Information), Lys‐AFs‐Ceria remained well‐dispersed after one month of preservation, while significant aggregates formed in both the CeO_2_ NPs and Lys‐Mon‐Ceria groups. A similar phenomenon was observed in the AFM images (Figure S11, Supporting Information), and this aggregation behavior resulted in the disappearance of the O_2_^•−^ elimination capacity. Surprisingly, Lys‐AFs‐Ceria maintained 50% SOD‐like activity compared with the freshly‐prepared samples (Figure S12, Supporting Information). This sustained activity can be attributed to the excellent dispersibility of Lys‐AFs, which prevented the formation of ceria nanozyme precipitates. Besides, the reducing capacity of Lys‐AFs might contribute avoiding the oxidation of surface ceria nanozymes.

The SOD‐CAT cascade catalysis of ceria nanozymes relies critically on the rapid transition between the Ce^3+^ and Ce^4+^ states.^[^ ^34^ ^]^ After the SOD activity assays, all samples underwent a subsequent XPS analysis. As exhibited in Figure S13 (Supporting Information), the ratio of Ce^3+^/Ce^4+^ decreased to 0.99 in Lys‐AFs‐Ceria and 0.56 in CeO_2_ NPs after SOD reaction. When the proportion of Ce^3+^ decreased, the CAT‐like activity of the ceria nanozyme became the predominant factor in the catalytic process. This shift was verified by evaluating CAT‐like activity through the Amplex Red probe method, where H_2_O_2_ acting as the substrates, were catalyzed into oxygen, and residual H_2_O_2_ was subsequently captured by Amplex Red probe to form resorufin with the typical absorbance at 571 nm (Figures S9 and S14, Supporting Information). As depicted in Figure 2j, 89.42±3.83% of H_2_O_2_ was decomposed in the Lys‐AFs‐Ceria group, whereas only 56.37±7.16% and 75.30±3.18% were decomposed in CeO_2_ NPs and Lys‐Mon‐Ceria. This superior CAT‐like activity in the Lys‐AFs‐Ceria group could be due to their better dispersion with the homogenous size, and a balanced Ce^3+^/Ce^4+^ ratio after the SOD‐like catalytic process. In addition, the CAT‐like activity of Lys‐AFs‐Ceria and CeO_2_ NPs demonstrated concentration‐dependent behavior (Figure S15, Supporting Information), and the activity of Lys‐AFs‐Ceria solution (including 1000 µM ceria) was comparable to standard catalase (160 U·mL^−1^), demonstrating the excellent CAT‐like catalytic activity of Lys‐AFs‐Ceria. The dissolved oxygen (DO) level was employed as another indicator of the CAT‐like activity, and with the Lys‐AFs‐Ceria group exhibiting the highest DO level at 18.80±0.36 mg mL^−1^, significantly superior to the CeO_2_ NPs group (14.23±0.35 mg·mL^−1^) (Figure 2k).

To reveal the CAT‐like enzymatic catalysis mechanism, we calculated the maximum reaction velocity (*V_max_*) and Michaelis–Menten constants (*K_m_*) via Michaelis‐Menten equation in H_2_O_2_ decomposition. As shown in Figure S16a,b (Supporting Information), a steady–state kinetic analysis was performed by changing the concentration of H_2_O_2_ (20, 50, 100, 200, and 400 mM) at a fixed concentration of CeO_2_ or Lys‐AFs‐Ceria, which was consistent with the classic Michaelis–Menten kinetic. According to the corresponding Lineweaver–Burk plots (Figure S16c,d, Supporting Information), the enzyme kinetic parameters were calculated, and the values of *K*_m_ and *V*_max_ were found to be 28.78±5.96 mM and 0.68 mg L^−1^·min^−1^ for the pristine CeO_2_ nanozymes at pH 7.4, whereas those of Lys‐AFs‐Ceria were 144.86±25.23 mM and 0.75±0.04 mg L^−1^·min^−1^ (Figure S16e, Supporting Information). While the elevated *K_m_* reflects reduced substrate affinity under in‐vitro conditions (28.78 mM for pure CeO_2_ vs. 144.86 mM for Lys‐AFs‐Ceria), largely attributed to confined space in Lys‐AFs, this parameter must be interpreted within the physiological context of diabetic wounds. Diabetic wounds are characterized by persistently high H_2_O_2_ concentrations exceeding 100 µM, where catalytic efficiency is dominated by *V*_max_ instead of substrate affinity. The increased *V*_max_ suggests a higher CAT catalytic activity and that is due to smaller sizes of ceria nanozyme on the surface of Lys‐AFs. Following the CAT activity tests, the Ce^3+^/Ce^4+^ ratio of Lys‐AFs‐Ceria showed an increase of Ce^3+^/Ce^4+^ ratio from 0.99 to 1.25, while CeO_2_ NPs group only exhibited a slight increase from 0.56 to 0.79 (Figure S13, Supporting Information), suggesting that Lys‐AFs‐Ceria still had enough surface oxygen vacancies for O_2_^•−^ scavenge. Encouraged by these results, we continued to measure the Ce^3+^/Ce^4+^ ratio of Lys‐AFs‐Ceria after five cycles of SOD‐CAT catalytic process, and found its ratio could remain at 0.99 (Figure S17, Supporting Information), suggestive of the better catalytic stability and durability than pristine CeO_2_ nanozyme. Our findings conclusively demonstrate that Lys‐AFs‐Ceria exhibits superior SOD‐CAT cascade catalytic activity compared to the CeO_2_ NPs group, emphasizing the critical role of amyloid fibril templates in synthesizing homogenous nanosized Ce^3+^‐rich nanoparticles, thus providing a solid rationale for their exceptional catalytic capabilities.

### 2.3 Fabrication and Characterization of Amyloid‐Templated Ceria Nanozyme Reinforced Hydrogel

**Figure 3:**
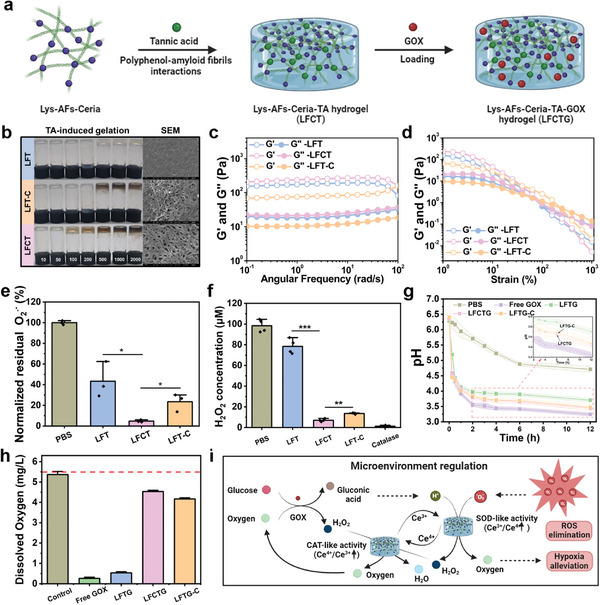
Morphology, rheology, and catalytic activity characterization of Lys‐AFs‐Ceria nanozyme‐reinforced hydrogel. a) The schematic illustration of Lys‐AFs‐Ceria nanozyme‐reinforced hydrogel preparation. This panel was created with www.biorender.com. b) Vial inversion tests of and SEM images of LFT (Lys‐AFs‐Tannic acid hydrogel), LFT‐C (Lys‐AFs‐Tannic acid hydrogel with CeO_2_ NPs), and LFCT hydrogels (Lys‐AFs‐Ceria‐Tannic acid hydrogel) via tannic acid‐induced gelation. The SEM images are taken from hydrogel with 2 mM tannic acid. c) Angular frequency‐dependent and d) strain‐dependent rheological measurements of different kinds of hydrogel. e) SOD activity assays (*n* = 3) of different samples including PBS, LFT, LFCT, and LFT‐C. f) Residual H_2_O_2_ concentration (CAT activity) assays (*n* = 4) of different samples (PBS, LFT, LFCT, and LFT‐C). Catalase serves as the positive control in CAT activity assays. g) The pH change (*n* = 3) and h) dissolved oxygen assays (*n* = 3) of glucose solution after catalysis of different GOX‐loaded hydrogels. i) Schematic representation of synergetic cascade catalysis process including glucose consumption mediated by GOX, and SOD/CAT catalytic reactions derived from Lys‐AFs‐Ceria nanozymes (left) and schematic diagram of synergistic cascade catalytic mechanism of LFCTG hydrogel in remodeling the adverse microenvironment of diabetic wounds (right). This panel was created with www.biorender.com. Statistical significance was defined as *p < 0.05, **p < 0.01 and ***p < 0.001.

Nanozymes have been extensively utilized in catalytic nanomedicine, particularly for modulating the challenging microenvironments of inflammatory diseases,^[^ ^35^ ^]^ such as, diabetic wounds. The catalytic performance of nanozymes largely depends on their sizes and dispersity. However, synthesizing ultrafine nanozymes and mitigating their tendency to agglomerate pose significant challenges. A recent example of amyloid‐mediated catalysis in vivo for alcohol detoxification demonstrates the potential advantages of using amyloid fibril as scaffolds,^[^ ^17^ ^]^ and provides a solid basis for amyloid‐templated ceria nanozyme synthesis. In this study, we expanded to develop a polyphenol‐amyloid fibril‐based hydrogel for the treatment of bacteria‐infected diabetic wounds, which are characterized by a tissue repair‐suppressing microenvironment with elevated levels of ROS and hypoxia. The amyloid‐templated ceria nanozyme was anticipated to function as a catalyst to remodel the infection microenvironment, converting elevated ROS into oxygen to alleviate hypoxia through SOD‐CAT cascade catalysis. The hydrogel form was found to be particularly suitable for infectious diabetic wound. Previous studies have demonstrated that gelation could be achieved through non‐covalent interactions between polyphenols and amyloid fibrils.^[^ ^15^, ^28^ ^]^ To optimize the gelation conditions, we initially tested three representative polyphenols including glycyrrhizic acid (GA), epigallocatechin gallate (EGCG), and tannic acid (TA), to trigger hydrogelation with Lys‐AFs‐Ceria. Among them, TA required the lowest concentration required for gelation (100 µM), significantly lower than GA (500 µM) and EGCG (200 µM) as demonstrated in vial inversion tests (Figure S18, Supporting Information), suggesting a stronger binding affinity between amyloid fibrils and TA (**Figure** **3**a). Interestingly, the gelation concentration required for TA in combination with Lys‐AFs‐Ceria was observed to be lower than that for Lys‐AFs and TA, suggesting that the presence of nanoceria on the surface of Lys‐AFs strengthened the non‐covalent interactions between them, leading to a reduced gelation concentration. Figure 3b displays the vial inversion and SEM images of three different hydrogel groups. The Lys‐AFs‐TA hydrogel (LFT) and Lys‐AFs‐TA hydrogel loaded with CeO_2_ NPs (LFT‐C) groups served as controls, while Lys‐AFs‐Ceria‐TA hydrogel (LFCT) was the experiment group. Comparatively, the LFCT group possessed lower gelation concentration and a sparser hydrogel network structure than the other two groups. Moreover, the mechanical strength of LFCT was higher than that of LFT and LFT‐C NPs, and the inclusion of CeO_2_ NPs slightly decreased the storage modulus of LFT (Figure 3c,d). Rheological experiments demonstrated that all hydrogel types could achieve gelation simply by mixing (Figure S19, Supporting Information), exhibiting excellent mechanical stability at the ultrahigh frequency (>100 rad·s^−1^) and strain (>100%), along with remarkable thixotropic properties (Figure S20, Supporting Information). More importantly, LFCT hydrogel exhibited the highest O_2_^•−^ elimination (95.3±1.18%) and H_2_O_2_ conversion (92.97±1.35%) capacity compared to LFT (SOD 56.7±17.21%, CAT 21.6±7.22%) and LFT‐C NPs (SOD 76.51±8.58%, CAT 86.47±0.6%) (Figure 3e,f). These findings demonstrated the promising stability and catalytic potential of these hydrogels for clinical applications and suggested that TA addition could enhance both the mechanical strength and catalytic activity of hydrogels. Besides, these results highlighted the advantages of amyloid‐templated ceria nanozymes in the improvements of catalytic activity compared with solely‐synthesized CeO_2_ NPs, evidenced by the differences between LFCT and LFT‐C hydrogel groups.

Elevated glucose levels can hinder angiogenesis and increase the risk of bacterial infections in diabetic wounds, ultimately complicating tissue repair.^[^ ^36^ ^]^ To address this, GOX was incorporated into the above hydrogels to prepare three variants: Lys‐AFs‐TA‐GOX hydrogel (LFTG), Lys‐AFs‐TA‐GOX hydrogel loaded with CeO_2_ NPs (LFTG‐C), and Lys‐AFs‐Ceria‐TA‐GOX hydrogel (LFCTG). In this configuration, glucose in diabetic wounds is oxidized by GOX to form gluconic acid and H_2_O_2_, leading to a pH decrease. As depicted in Figure 3g, the pH of the glucose solution serving as the negative control, decreased from 6.5 to 5.0 due to the background oxidation in the water buffer. By contrast, free GOX, serving as the positive control, exhibited the optimal catalytic performance, reducing the pH to 3.3. The pH of the glucose solution experienced a rapid decrease from 6.5 to 4.0 within the initial 2 h following the addition of various hydrogels, subsequently stabilizing at 3.5 after 12 h. This implies that GOX retained its catalytic activity within the hydrogels. The synergistic actions of GOX‐mediated glucose consumption and the SOD‐CAT cascade catalysis by Lys‐AFs‐Ceria were anticipated to transform ROS and glucose into oxygen. To evaluate their synergistic effect, the dissolved oxygen (DO) level in the glucose solution at 0 h and 12 h was measured (Figure 3h). After 12 h, both the free GOX and LFTG groups exhibited extremely low DO levels, suggesting that GOX‐mediated glucose oxidation effectively depleted the initial DO content, without ceria, missing the process of transforming H_2_O_2_ into O_2_ and H_2_O. In contrast, the LFCTG and LFTG‐C hydrogels, due to the inclusion of ceria nanozyme, maintained a sufficient DO level even after 12 h. CAT‐mimetic activity of ceria nanozymes generally decreases significantly under acidic conditions, and this is because ceria nanozymes rely on the cycling between Ce^3^⁺ and Ce⁴⁺ oxidation states to retain CAT activity, which is optimal around neutral to mildly alkaline pH levels. To further prove the influence of acidic microenvironment form GOX on CAT‐mimetic activity of LFCTG hydrogel, the CAT‐mimetic activity assays were performed in various pH conditions (pH = 3.5, 4, and 7), and the results proved that LFCTG hydrogel exhibited similar CAT activity in acidic pH and in neutral pH conditions as shown in Figure S21 (Supporting Information), which might be associated with the reducing activity^[^ ^11^ ^]^ and the stabilization of active sites^[^ ^17^, ^37^ ^]^ provided by the Lys‐AFs, enhancing the redox cycling of Ce^3+^/Ce^4+^ and stabilizing of catalytic sites. These findings confirmed the remarkable synergistic cascade catalytic activity of glucose consumption catalyzed by GOX and the SOD‐CAT catalysis exhibited by Lys‐AFs‐Ceria. This combination significantly achieved ROS elimination and hypoxia alleviation, effectively remodeling the adverse microenvironment of diabetic wounds (Figure 3i).

### 2.4 Bioactivities Characterization of Amyloid‐Templated Ceria Nanozyme Reinforced Hydrogel

Prior to evaluating the intracellular catalytic capabilities, the cytotoxicity of various hydrogels on fibroblasts (L‐929) was assessed using the methyl thiazolyl tetrazolium (MTT) assay. Figure S22 (Supporting Information) demonstrate that all hydrogels and most hydrogel components were non‐toxic to L‐929 cells at operational concentration, except GOX (0.5 mg·mL^−1^) and TA (2 mM). Surprisingly, the cytotoxicity induced by GOX and TA decreased dramatically after hydrogel formation, and the cytotoxicity of hydrogel decreased as the TA concentration increased in the LFCTG hydrogel. When the TA concentration reached 1.5 and 2 mM, the hydrogel exhibited excellent cytocompatibility (Figure S23, Supporting Information). The enhanced hydrogel mechanical strength with the increased TA concentration might stabilize GOX and TA, thus reducing their cytotoxicity. Similar results were also observed in hemocompatibility assays, where the hemolysis of 2 mM TA exceeded 5% yet still showed good hemocompatibility after hydrogel formation (Figures S24 and S25, Supporting Information). As a result, the optimal TA concentration was set at 2 mM for the subsequent experiments unless otherwise specified.

**Figure 4:**
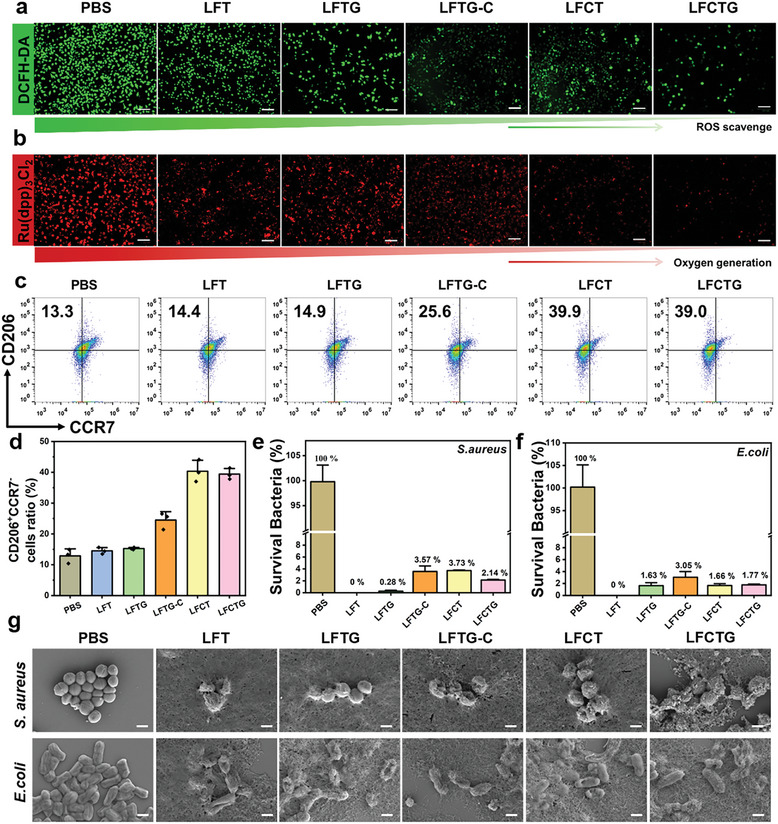
Bioactivities characterization of amyloid fibrils‐templated ceria nanozyme‐reinforced hydrogel. a) The alleviation of oxidative stress in L‐929 cells was monitored via a ROS probe (DCFH‐DA) after different hydrogel treatments. Scale bar: 100 µm. b) Intracellular O_2_ generation assay was validated by hypoxic probe Ru(dpp)_3_Cl_2_ after different treatments of L‐929 cells. (Scale bar: 100 µm) c) Representative flow cytometry analysis images and d) quantitative results (*n* = 3) of Raw 264.7 cells polarization behaviors after incubation with different hydrogel samples. Antibacterial activity of different hydrogels against e) *S. aureus* (*n* = 3) and f) *E.coli* (*n* = 3). g) SEM images of *S. aureus* and *E.coli* strains after treatments of different hydrogels. (Scale bar: 500 nm).

We further explored the intracellular ROS elimination activity of the hydrogel. The green fluorescence of L‐929 cells increased significantly following the 100 µM H_2_O_2_ treatment (**Figure** **4**a), revealing the successful establishment of in‐vitro ROS model.^[^ ^38^ ^]^ Compared with the control group, the fluorescence intensity of the LFCTG and LFCT groups exhibited notable reductions, which were more pronounced than that of the LFCT‐C group, indicating that the amyloid‐templated ceria nanozyme possessed higher ROS elimination capacity. Subsequently, hypoxic probe Ru(dpp)_3_Cl_2_ was employed to monitor the intracellular hypoxia level, where a weaker red fluorescence intensity indicated higher oxygen content.^[^ ^39^ ^]^ Similarly, 100 µM H_2_O_2_ treatments elicited a severe hypoxic condition with intense red fluorescence. However, significant improvements in hypoxia were observed across all groups following the incorporation of various hydrogels (Figure 4b). These improvements were partially attributed to the antioxidant activity of TA and partially to the catalytic activity of the ceria nanozyme. Notably, the red fluorescence intensity of the LFCTG and LFCT groups was significantly lower compared to the LFCT‐C group, indicating that the Lys‐AFs templated ceria nanozyme exhibited greater catalytic activity in mitigating hypoxia. These findings suggested that as‐prepared hydrogel could induce the transformation of intracellular ROS to oxygen via the cascade catalysis, and may benefit the treatment of diabetic wounds.

Further in‐vitro studies explored additional bioactivities of the hydrogel, such as promoting cell proliferation, regulating macrophage polarization, and enhancing antibacterial activity, essential for diabetic wound healing. The design concept of the hydrogel integrated lysozyme amyloid fibrils, which were anticipated to enhance cell proliferation.^[^ ^3^, ^12^ ^]^ Amyloid fibrils inherently facilitate cell adhesion, a property consistent across various sequences and modifications.^[^ ^13^, ^28^ ^]^ Previous research has demonstrated that Lys‐AFs can facilitate cell adhesion, spreading, and proliferation. In this work, cell proliferation experiments were conducted using the MTT method and documented with confocal laser scanning microscopy (CLSM). Upon incubating L‐929 cells with different hydrogel groups for 48 h, an increase in cell number was observed across all groups, suggesting the vital role of Lys‐AFs in cell proliferation (Figure S26, Supporting Information). Moreover, L‐929 cells began spreading and exhibited a fusiform appearance within the first 12 h in all hydrogel‐treatment groups, whereas cells in the control group largely retained a circular morphology. In fact, this spreading and focal adhesion complexes suggest a direct connection between the cells and hydrogels.^[^ ^8a^ ^]^ In‐vitro angiogenesis promotion activity of hydrogels was also evaluated through TRITC Phalloidin‐staining tube formation assays using HUVEC cells. As shown in Figure S27 (Supporting Information), LFCTG hydrogel group showed the highest tube formation amount and length compared with other control groups, indicating LFCTG hydrogel significantly promoted angiogenesis in‐vitro and the oxidative stress microenvironment improvement was essential for the promotion of angiogenesis. Besides, our ceria nanozyme‐reinforced hydrogels (LFTG‐C, LFCT, and LFCTG) induced macrophage polarization from M1‐type to M2 type facilitated by the presence of Ce ions (Figure 4c,d). The LFCT and LFCTG hydrogels showed greater regulatory activity on macrophage polarization over the LFTG‐C group due to the great dispersity and stability provided by the amyloid fibril templates.

Additionally, our Lys‐AFs hydrogels also exhibit high antibacterial activity. Monomeric lysozyme is well known for its bactericidal activity by hydrolyzing the peptidoglycan of the cell wall in Gram‐positive bacteria.^[^ ^40^ ^]^ However, Gram‐negative bacteria are less susceptible to lysozyme activity due to their outer membrane, which impedes lysozyme penetration. Unlike monomeric lysozyme, Lys‐AFs are formed through the hydrolysis, unfolding, and assembly of lysozyme monomers, displaying broad‐spectrum antibacterial activity against both Gram‐positive and Gram‐negative bacteria via a totally different mechanism. The ordered structure of amyloid fibrils with cationic, hydrophobic, and conformational properties is believed to contribute to bacterial aggregation and killing while maintaining biocompatibility.^[^ ^15a^ ^]^ In this work, *Staphylococcus aureus* (*S. aureus*) and *Escherichia coli* (*E. coli*) were selected as the representative Gram‐positive and Gram‐negative strains to further assess the antibacterial activity of the Lys‐AFs based hydrogel. When checking the survival rate of *S. aureus* and *E. coli* (10^7^ CFU mL^−1^) after the treatments of different groups for 8 h, exceptional antibacterial performances were observed in LFT (100% *S. aureus* and 100% *E. coli*) and LFTG (99.72±0.14% *S. aureus* and 98.37±0.52% *E. coli*), suggesting an enhanced antimicrobial activity by combination of Lys‐AFs and TA (Figure 4e,f; Figure S28a, Supporting Information). Surface‐modified ceria nanozyme appeared to partially hinder direct contact between bacteria and Lys‐AFs, resulting in a moderate decrease in antibacterial performance.^[^ ^15^, ^28^, ^41^ ^]^ However, these hydrogels still exhibited excellent broad spectrum bactericidal performance without the preference for bacterial types, such as LFCT (96.27±0.10% *S. aureus* and 98.34±0.31% *E. coli*), LFCTG (97.86±0.11% *S. aureus* and 98.23±0.14% *E. coli*), and LFCT‐CeO_2_ NPs (96.43±0.93% *S. aureus* and 96.95±0.95% *E. coli*). Such antibacterial performances were enough for diabetic wound managements. SEM images (Figure 4g) and SYTO9/PI staining results (Figure S28b, Supporting Information) showed that the surfaces of bacteria become crumpled, distorted, and even cracked after hydrogel treatment in comparison to the control group, highlighting that the efficacy of antibacterial activity depends on the direct contact between hydrogel and bacteria, potentially via membrane‐disruption mechanism. Collectively, the ceria nanozyme‐reinforced hydrogels exhibited outstanding biocompatibility and were endowed with multiple bioactivities, including cascade catalytic activity, cell proliferation acceleration, macrophage regulation, and antibacterial activity. These properties fulfilled all the essential requirements for diabetic wound healing.

### 2.5 Fabrication and Characterization of an Amyloid‐Templated Ceria Nanozyme Reinforced Microneedle

**Figure 5:**
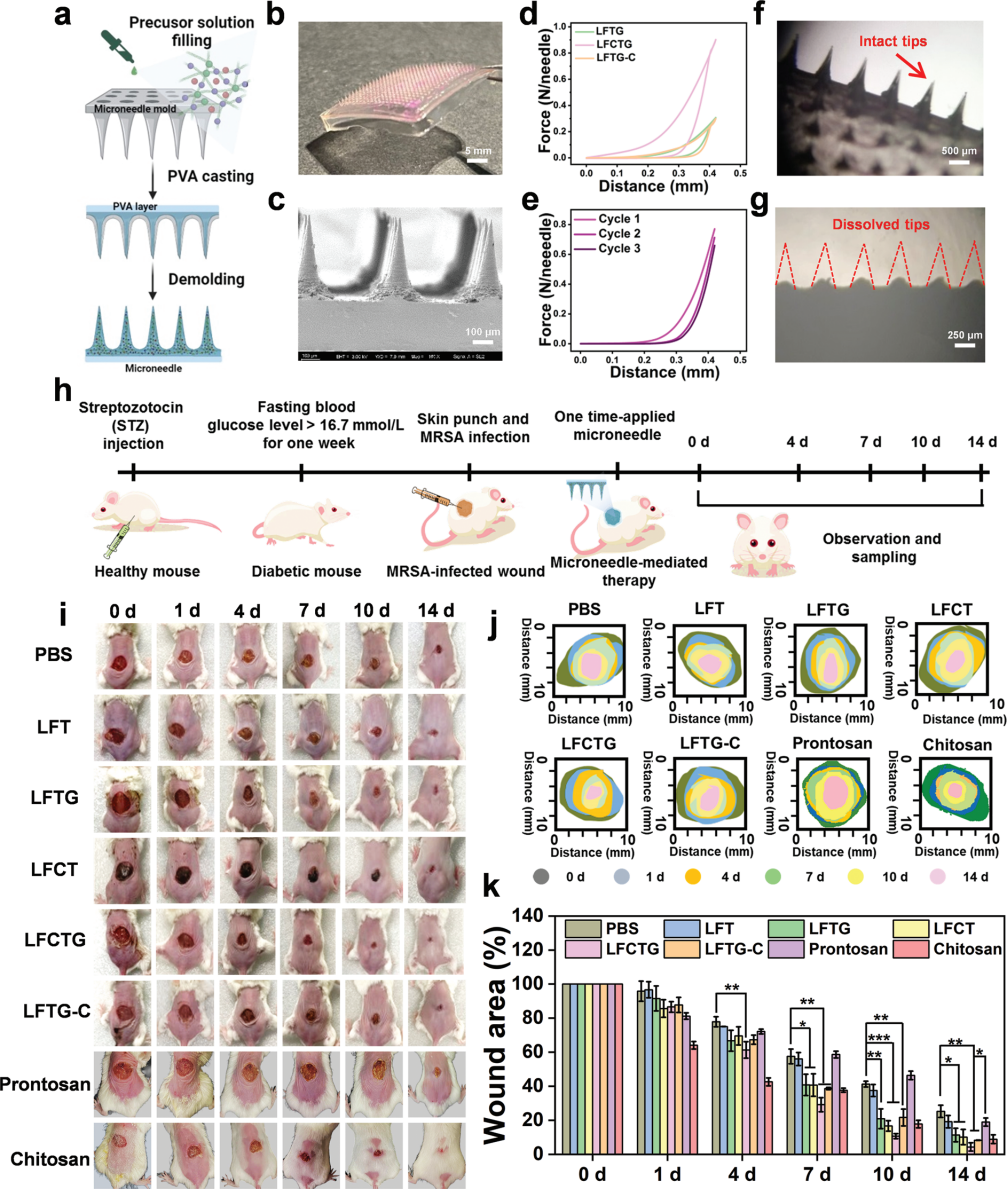
Preparation and characterization of amyloid‐templated ceria nanozyme reinforced microneedle and in vivo studies of microneedle therapy. a) The schematic representation of LFCTG‐based microneedle fabrication. b) Representative pictures (Scale bar: 5 mm) and c) SEM images of LFCTG‐based microneedle (Scale bar: 100 µm). d) Mechanical compression force curves of different hydrogel‐based microneedles including LFTG, LFCTG, and LFTG‐C. e) Mechanical compression force curves of LFCTG‐based microneedles during three cycles. Picture of microneedle patch f) before (Scale bar: 500 µm) and g) after (Scale bar: 250 µm) applied on the mouse skin for 2 h. Red row indicated the intact microneedle tips, while dotted line showed the dissolved microneedle tips. h) Schematic illustration of building model of MRSA‐infected diabetic mice and the process of microneedle‐treatments. This panel was created with www.biorender.com. i) Representative images of diabetic wounds under MRSA infection at day 0, 1, 4, 7, 10, and 14. j) The schematic diagram of wound trace in the healing process from i. k) Quantification of wound closure ratio (*n* = 5) in diabetic mice under infection at day 0, 1, 4, 7, 10, and 14 for all groups including PBS, LFT, LFTG, LFCT, LFCTG, LFTG‐C, Prontosan hydrogel and Chitosan hydrogel. Note: *p<0.05, **p<0.01, and ***p<0.001 versus PBS‐treated group.

To improve the practicality of these hydrogels in diabetic wound management, amyloid‐templated ceria nanozyme‐reinforced microneedles were fabricated for in vivo studies. The concept of “microneedles” was proposed in 1976 to facilitate drug delivery with the advantage of penetrating the skin and overcoming physiological barriers.^[^ ^42^ ^]^ Briefly, the hydrogel precursor solutions were filled into the cavities of the microneedle patch molds (20×20 arrays) to form the microneedle tips, while the base of the microneedle patch was composed of a 20 wt.% polyvinyl acetate (PVA) solution (**Figure** **5**a). These microneedles have transparent textures and notable flexibility (Figure 5b; Figure S29a,b, Supporting Information), featuring microneedle tips with a height of 600 µm and a base diameter of was 250 µm, arranged in a periodic space of 550 µm (Figure 5c; Figure S30, Supporting Information). Previous studies demonstrated that the skin penetration standard of force was above 0.098 N/microneedle tips.^[^ ^43^ ^]^ In the mechanical compression force tests, all three types of microneedles met this criterion, with LFCTG microneedle demonstrating the most robust mechanical properties (0.90 N needle^−1^) compared to the LFTG and LFTG‐C microneedles (Figure 5d). An increasing TA concentration further enhanced the mechanical strength of the LFCTG microneedle (Figure S29c, Supporting Information). These results aligned with the previously noted rheological characteristics of their corresponding hydrogels (Figure 3c,d). Furthermore, microneedles withstood three‐cycle measurements while retaining stable mechanical strength (Figure 5e). Eventually, these amyloid‐templated ceria nanozyme‐reinforced microneedles were applied to the back skin of mice for wound management (Figure S29d, Supporting Information) and the microneedle tips were observed to dissolve after 2 h retention (Figure 5f,g). After the removal of microneedles, visible micropores were evident on the skin surface of mice (Figure S29e, Supporting Information). This deliberate design of microneedles contributes to achieve a two‐stage release of active components for the treatment of infected diabetic wounds. First, the rapid dissolution of microneedles ensures efficient delivery of hydrogel matrix (Lys‐AFs‐Ceria, GOX, and TA) into deep wound tissues, bypassing the epidermal barrier. This is critical for addressing infections and oxidative stress in hypoxic regions. Subsequently, the residual hydrogel matrix forms a bioactive reservoir that gradually releases active components post dissolution. To our knowledge, this is the first demonstration of amyloid fibrils‐based microneedles where the tips were completely independent of commercial materials. Our findings pave the way for the in vivo treatments of diabetic wounds.

### 2.6 Bacteria‐Infected Wound Model of Diabetic Mice for in vivo Characterization

The therapeutic efficiency of the as‐prepared microneedles for the diabetic wounds was evaluated in vivo. A bacteria‐infected wound model of diabetic mice was established as shown in Figure 5h. The blood glucose levels of mice maintained above 11 mM for one week, confirming the successful construction of the diabetic model.^[^ ^44^ ^]^ Then, oval full‐thickness wounds with a diameter of 10 mm were created on the backs of the diabetic mice, and 100 µL bacterial suspensions at a concentration of 10^8^ CFU mL^−1^ were inoculated on the wounds for 1 day to form wound infections. All mice were divided into 8 groups for 12 h separately, including untreated group and five kinds of microneedle patches made of LFT, LFTG, LFCT, Protosan hydrogel, Chitosan hydrogel, LFCTG, and LFTG‐C. Wound progression was monitored daily over 14 days. Figure 5i–k show images of the wounds, wound traces, and the corresponding quantified wound area data at various time points. The wound closure rate of untreated group was 74.75±3.58% after 14 days, whereas the LFCTG microneedle patch exhibited the highest wound closure, exceeding 95.71±2.46%. The other groups also demonstrated varying degrees of wound closure, ranging from 80.89±3.61% to 91.81±0.15%.

**Figure 6:**
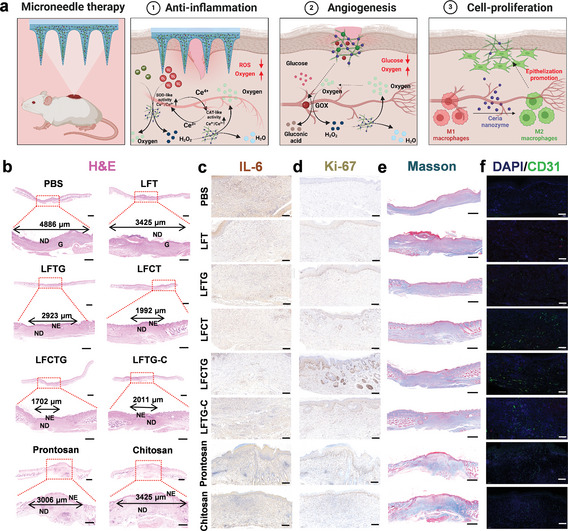
Slice analysis of diabetic wound after treatments of different kinds of microneedle. a) Schematic illustration of the anti‐inflammation, angiogenesis, cell proliferation mechanisms of microneedle therapy in diabetic wound. This panel was created with www.biorender.com. Representative b) H&E, c) IL‐6, d) Ki‐67, e) Masson, f) CD31 (green) immunofluorescence staining analysis of the skin tissues of MRSA‐infected diabetic mice wounds after different treatments including PBS, LFT, LFTG, LFCT, LFCTG, LFTG‐C, Prontosan hydrogel and Chitosan hydrogel. In H&E slices, G represents granulation tissue, NE represents newly formed epidermis, and ND represents newly formed dermis. These rows indicate the scale of wound. Scale bar: 2 mm (top), 200 µm (below). The scale bar of Masson slices (e) is 200 µm. The scale bars of (c), (d), and (f) are 100 µm.

Elevated ROS and glucose level significantly prolong the healing process of diabetic wounds, rendering them particularly vulnerable to bacterial infections due to long‐term exposure. In addition, high glucose concentration obstructs the formation of new blood vessels, exacerbating the hypoxia conditions within the wound microenvironment.^[^ ^41^ ^]^ Our newly developed amyloid‐templated ceria nanozyme reinforced microneedles are designed to improve the adverse microenvironment (anti‐inflammatory) and acceleration of diabetic wound healing (angiogenesis and cell proliferation) through the synergistic effect of GOX‐mediated glucose consumption and ceria nanozyme‐involved SOD‐CAT cascade catalysis, coupled with multiple bioactivities from amyloid fibrils (**Figure** **6**a). Hemotoxylin and eosin (H&E) staining confirmed the superior performance of LFCTG microneedle, which facilitated the smallest wound size and complete epidermis/dermis regeneration (Figure 6b). The formation of a thorough new epidermis (NE) was also observed in the LFCT, LFCTG, and LFTG‐C groups, highlighting the therapeutic efficacy of ceria nanozyme for diabetic wounds. Notably, the enhanced performance of the LFCTG over the LFTG‐C highlights the advantages of using the Lys‐AFs template to boost the catalytic activity of ceria nanozymes, promoting glucose consumption facilitated by GOX and ROS, thereby emphasizing the unique significance of each component within the microneedles.

To further investigate improvements in the microenvironment of diabetic wounds, we analyzed skin tissue samples obtained from wound sites, focusing particularly on inflammation, cell proliferation, collagen formation, and angiogenesis. IL‐6, a well‐established pro‐inflammatory cytokine, is significantly elevated in response to microbial infections and inflammation. Figure 6c depicts the sustained high expression of IL‐6 in the control group, suggesting either incomplete infection elimination or a persistent and hyperactive inflammatory response despite the evidence of dermal regeneration. Notably, the LFCTG microneedle treatments displayed a higher reduction of inflammation than other groups (including Protosan and Chitosan hydrogel), as confirmed by the decreased IL‐6 expression. To further demonstrate the ROS‐scavenging and antibacterial capabilities of our microneedles, we performed dihydroethidium (DHE, a ROS probe) and Giemsa staining (for live bacteria) analysis of skin tissues with wounds. As shown in Figure S31 (Supporting Information), the presence of ceria nanozyme in our microneedles significantly decreased ROS levels in diabetic wounds, as seen in the LFCT, LFTG‐C and LFCTG groups, while stronger ROS signals persisted in the PBS (negative control) and both positive control groups. Giemsa staining showed nearly no live bacteria in the LFCTG group, which also suggested that bacterial infection might be a major contributor to the elevated ROS levels observed in diabetic wounds. These results confirm that our microneedles provide effective ROS scavenging and antibacterial activity at the wound site. Concerning tissue regeneration in diabetic wound, LFCTG microneedle treatment group exhibited the highest levels of expression for Ki‐67 (Figure 6d) and comparable collagen deposition (Figure 6e) with other groups. Collagen fibrils, important markers of extracellular matrix regeneration at the wound site, underscore the role of Lys‐AFs in promoting cell proliferation and collagen regeneration. Similarly, the most obvious angiogenesis phenomenon was observed in LFCTG microneedle treatment group, evidenced by the highest expression level of CD31, VEGFA, and HIF‐1α (Figure 6f; Figure S32, Supporting Information) among all groups. These groups with ceria nanozyme (including LFCT, LFTG‐C, and LFCTG) exhibited more obvious angiogenesis phenomena, while a relatively lower expression of CD31, VEGFA, and HIF‐1α was observed in control groups, including PBS, Prontosan hydrogel, and Chitosan hydrogel group, demonstrating that the oxidative stress microenvironment remodeling was essential for angiogenesis at the diabetic wound sites. Combined with in‐vitro angiogenesis results, both the cell proliferation capacity from Lys‐AFs and the oxidative stress microenvironment remodeling provided by ceria nanozymes are essential for angiogenesis promotion in the diabetic wounds. In brief, LFCTG microneedle patches effectively mitigated inflammation and facilitated cellular proliferation and angiogenesis in MRSA‐infected diabetic wounds. The reduction of localized high glucose concentrations at the wound sites, combined with ROS clearance, contributes significantly to cell proliferation and angiogenesis. Moreover, all groups of the mice still maintained stable body weights (Figure S33, Supporting Information) throughout 14‐day of in vivo experiments, and blood biochemistry analysis revealed normal levels of biochemical markers, including alanine aminotransferase (ALT), aspartate aminotransferase (AST), alkaline phosphatase (ALP), and blood urea nitrogen (BUN) (Figure S34, Supporting Information), indicating the preservation of normal liver and kidney functions. Additionally, histological analysis of major organs from the sacrificed mice showed no observable damage or lesions (Figure S35, Supporting Information). These results demonstrate the excellent in vivo biosafety of our microneedles, reinforcing their potential for further applications.

## 3 Conclusions

In summary, we have successfully synthesized ceria nanozymes with enhanced SOD‐CAT catalytic activity and prolonged stability through an amyloid fibrils‐template strategy. These nanozymes have been effectively employed in the development of microneedles tailored for the managing diabetic wounds. The ordered structure and surface chemistry of amyloid fibrils facilitated the binding of Ce ions and the in situ growth of ceria nanozymes, resulting in homogenous nanosized ceria nanozymes with an elevated Ce^3+^/Ce^4+^ ratio and suppressing aggregation propensity. These properties are crucial for mitigating the elevated oxidative stress and severe hypoxia commonly observed in diabetic wounds. As a demonstration of application, we developed amyloid fibrils‐templated ceria nanozyme‐reinforced hydrogels and subsequently used them to design a microneedle by incorporating TA and GOX. These hydrogels displayed excellent biocompatibility and effectively regulated the infection microenvironment through GOX‐mediated glucose consumption and the cascade catalysis of ceria nanozymes, leading to the efficient transformation of high levels of ROS and glucose into oxygen. Furthermore, these hydrogels exhibited remarkable antibacterial properties and promoted cell proliferation. in vivo experiments demonstrated that the fabricated Microneedle significantly expedited the healing of diabetic wounds by reducing inflammation, enhancing cell proliferation, and promoting angiogenesis. This work presents a promising strategy for the synthesis of ceria nanozymes with superior catalytic activity and stability, while introducing an original amyloid fibrils‐based microneedle with transformational potential for the treatment of bacterial infections in diabetic wounds.

## 4 Experimental Section

### Preparation of Lysozyme Amyloid Fibrils (Lys‐AFs)

The preparation of lysozyme amyloid fibrils was according to previous reports.^[^ ^15b^ ^]^ Lysozyme powder (200 mg) was dissolved in 10 mL MilliQ water with thorough stirring, and then the pH value of the solution was adjusted to 2.0 using 1 M HCl. After that, the solution was heated in an oil bath at 90°C for 24 h at a stirring rate of ≈500 rpm min^−1^. The reaction terminated in an ice bath and the formation of amyloid fibrils was verified by birefringence under a polarized light microscope. The concentration of resulting solution is 2 wt.%, and named lysozyme amyloid fibrils (Lys‐AFs).

### Amyloid Fibrils‐Templated In Situ Synthesis of Ceria Nanozyme (Lys‐AFs‐Ceria)

The main step for the formations of amyloid fibrils‐templated in situ synthesis of ceria nanozyme followed closely and were inspired by previous reports in the production of iron nanoparticles‐decorated amyloid fibrils.^[^ ^11^ ^]^ 2.25 mL above 2 wt.% Lys‐AFs solution and 7.34 mL MilliQ water were mixed and stirred at 250 rpm min^−1^ for 5 min. Then, 0.15 mL 1 M Ce (NO_3_)_3_•6H_2_O solution was added drop by drop, and the resulting solution was stirred at 250 rpm min^−1^ for 30 min. After adding 0.26 mL 1 M NaBH_4_ solution slowly into above solution, the resulting solution was further stirred at 250 rpm min^−1^ for 30 min. In this process, CeO_2_ nanoparticles would be synthesized on the surface of Lys‐AFs via the reduction mechanism, and the solution obtained by this group is named Lys‐AFs‐Ceria. Similar with above procedure, another two control samples were also prepared. The preparation of pure cerium dioxide nanoparticle (CeO_2_ NPs) group was as followed: 0.15 mL 1 M Ce (NO_3_)_3_•6H_2_O solution and 9.59 mL MilliQ water were mixed and stirred at 250 rpm min^−1^ for 30 min. Then, 0.26 mL 1 M NaBH_4_ solution was slowly added and the resulting solution further stirred at 250 rpm min^−1^ for 30 min until the solution turns dark yellow. The solution obtained in this group was named CeO_2_ NPs. Synthesis of cerium oxide nanozyme (Lys‐Mon‐Ceria) by lysozyme monomer template was similar with the above process with the substitution of amyloid fibrils to amyloid monomers. The solution obtained in this group was named Lys‐Mon‐Ceria. When the controls (CeO_2_ NPs and Lys‐Mon‐Ceria) and experimental group keep the identical reaction conditions (same reductant concentration, pH, and temperature), it could better isolate the advantages of Lys‐AFs as the templates. Prior to hydrogel preparation, the above solutions were dialyzed with a dialysis membrane (MD34, Mw = 7000 Da) for 2 days to remove protein monomers, the unbinding nanozymes, and excess reductants. The high purification rate was assured by daily batch change.

### Hydrogel Preparation

The preparation of amyloid fibrils‐based hydrogel was according to previous reports.^[^ ^15^, ^28^ ^]^ 180 µL above solution (Lys‐AFs‐Ceria) was mixed with 20 µL tannic acid solution with/without dissolved 5 mg mL^−1^ GOX to form hydrogel overnight. The resulting hydrogel was termed as Lys‐AFs‐Ceria‐GOX‐TA hydrogel (LFCGT) and Lys‐AFs‐Ceria‐TA hydrogel (LFCT). Vial upside down experiments were performed to confirm the formation of hydrogel. Of note, the concentration of tannic acid was set as 5, 10, and 20 mM. Other control hydrogel samples, including Lys‐AFs‐TA hydrogel (LFT), Lys‐AFs‐GOX‐TA hydrogel (LFGT), Lys‐AFs‐T hydrogel with CeO_2_ NPs (LFT‐C), and Lys‐AFs‐TA‐GOX hydrogel with CeO_2_ NPs (LFGT‐C), were prepared using similar way. Among, LFT‐C hydrogel was prepared using the physical mixture of Lys‐AFs, tannic acid, and solely‐synthesized CeO_2_ NPs, and LFGT‐C hydrogel was prepared using the physical mixture of Lys‐AFs, tannic acid, GOX, and solely‐synthesized CeO_2_ NPs. Owing to pH difference between Lys‐AFs and Lys‐AFs‐Ceria solution, the pH of Lys‐AFs used in control groups should be adjusted into ≈5.8 using Bis‐tris buffer (pH = 6.8) before the preparation of hydrogel. It was noted that the final concentration of lysozyme monomer was 0.4 wt.% in all hydrogel samples.

### Microneedle Fabrication and Characterization

The microneedle patch molds (20×20 needles per microneedle patch) were bought from Engineering for Life Co. Ltd. China. The height of microneedle height was 600 µm and the spacing between tips was 550 µm. First, 400 µL hydrogel precursor solutions were filled into the cavities of the mold and then these molds were placed in a vacuum degassing device to remove bubbles. Next, these molds were placed in a drying oven at 35 °C for 12 h for the first casting. The above process was repeated for the second casting. Finally, 200 µL of PVA solution with 20 wt.% concentration was cast onto the bottom of microneedle patch. The microneedle patch was demolded and obtained after drying. The mechanical compression tests were conducted using a universal material testing machine (MTS CMT6103) to characterize the microneedle. The microneedle patch was placed on the specimen holder, and the mechanical sensor was set to approach the needle tip at a speed of 1 mm ^−1^s. When sensors contacted with the top of the microneedle tip, the approaching speed of the sensor was reduced to 0.2 mm ^−1^s, and the testing displacement of the sensor was set as 420 µm. Throughout the process, the displacement of the microneedle and the corresponding force were continuously measured and recorded. The cyclic mechanical tests were conducted in parallel three times. Besides, the skin penetration experiments were performed on skin of mouse back. The microneedle patch was pressed into the mouse skin, and kept for 10 min. Then, the microneedle patch was removed, observed, and recorded. The microscopic region of skin tissue was also recorded using camera.

### In Vivo Studies

All animal experiments were conducted in accordance with Chinese legislation on the Use and Care of Research Animals (Document No. 55, 2001), and institutional guidelines for the Care and Use of Laboratory Animals established by the Shanghai University Animal Studies Committee, and this committee approved the experiments (YS 2024–267). Streptozocin (STZ) powder was dissolved in the sodium citrate‐citric acid buffer (pH = 4.2) to prepare 10 mg mL^−1^ STZ solution. For the fabrication of diabetic wound model of mouse, BALB/c mice (with an average weight of 17 g) were fasted overnight, prior to in vivo experiments. Next day, STZ solution was injected intraperitoneally at a dosage of 150 mg STZ per kg of mouse body weight. After the injection, the mice's body weight and blood glucose levels were monitored and recorded during the five‐day normal feeding. When their blood glucose levels maintained above 11 mM, the construction of diabetic wound model of mouse was successful. Then, the diabetic mice were anesthetized with 1% pentobarbital sodium and circular cutter was used to create an oval full‐thickness wound with 10 mm diameter in the back of the mice. Immediately, 100 µL bacterial suspensions with the concentration of 10^8^ CFU mL^−1^ were inoculated on the wound for 1 d to form wound infection. All mice were divided into 8 groups and microneedle patch made of different kinds of hydrogel including LFT, LFTG, LFCT, LFCTG, Protosan hydrogel, Chitosan hydrogel, and LFTG‐C, were applied on the wounds using gentle pressure for 10 min. In addition, PBS treatment was set as the control group. 3 M wound dressings were used to secure the patches in place for 12 h. After that, the microneedle patches were removed and the infection status of the wounds was recorded daily for 14 days. Finally, these mice were euthanized and the skin tissue of the wounds was removed and fixed using 4% paraformaldehyde for histological analysis. Besides, the blood and main organs of diabetic mice containing heart, liver, spleen, lung, and kidney were taken out and for in vivo biosafety analysis.

### Statistical Analysis

The data were expressed as mean ± standard deviation (S.D). Statistical significance was analyzed by one‐way ANOVA and Student's t‐test using GraphPad Prism7 software. Statistical significance was defined as *p < 0.05, **p < 0.01, and ***p < 0.001. Each test in this study has no least than three (*n* ≥ 3) parallel samples for statistical analysis, and the notes of special labels or sample numbers were all given in related experiments.

## Conflict of Interest

The authors declare no conflict of interest.

## Author Contributions

Q.X., C.C., and R.M designed the study. Q.X., J.C., and X.Q. performed the experiments. J.C. and Y.G. performed antibacterial assays and intracellular ROS/DO assays. T.J. performed SEM experiments. J.Z. performed AFM characterization. Q.S. performed rheological experiments. L.Z. performed nanopore tests. Q.X., X.Q., M.P., B.L. C.C., H.L., P.W., and R.M analysed the results and Q.X. wrote the manuscript. All authors participated in the revision of manuscript. Q.X., H.L., C.C., P.W., and R.M supervised the project and provided the funding.

## Supporting information



Supporting Information

## Data Availability

The data that support the findings of this study are available from the corresponding author upon reasonable request.
