# Supplementary material for: Amyloid‐Templated Ceria Nanozyme Reinforced Microneedle for Diabetic Wound Treatments
Source: Adv Mater. 2025 Feb 25;37(15):2417774. doi: 10.1002/adma.202417774 (PMC12004906; doi:10.1002/adma.202417774)
Supplement: Supplementary file 1 — Supporting Information [file ADMA-37-2417774-s001.docx]

Supporting Information

**Amyloid-Templated Ceria Nanozyme Reinforced Microneedle for Diabetic Wound Treatments**

*Qize Xuan^1,2,3^, Jiazhe Cai^1^, Gao Yuan^1^, Xinchi Qiao^1^, Tonghui Jin^2^, Mohammad Peydayesh^2^, Jiangtao Zhou^2^, Qiyao Sun^2^, Lijian Zhan^5^, Bin Liu^2^, Ping Wang^3,4^, Hui Li^1^, Chao Chen^1,3^*, Raﬀaele Mezzenga^2,6^**

^1^ Institute for Environmental Pollution and Health, School of Environmental and Chemical Engineering, Shanghai University, Shanghai, 200444, PR China

^2^ Department of Health Sciences and Technology, ETH Zürich, Schmelzbergstrasse 9, Zürich 8092, Switzerland

^3^ State Key Laboratory of Bioreactor Engineering Center, School of Biotechnology, East China University of Science and Technology, Shanghai 200237, China

^4^ Department of Bioproducts and Biosystems Engineering, University of Minnesota, St Paul, MN 55108, USA

^5^ Institute for Biomedical Engineering, ETH Zürich, 8092 Zürich, Switzerland

^6^ Department of Materials, ETH Zurich, Wolfgang-Pauli-Strasse 10, 8049 Zürich, Switzerland

* Corresponding authors: chaochen@shu.edu.cn; raﬀaele.mezzenga@hest.ethz.ch

**Supplementary Experimental Section**

*Materials characterization:* For characterization, all solution samples need to perform dialysis treatments. The dialysis treatments were performed in the 22 mm (MD34) [M_W_=7000] dialysis bag *via* 200 rpm/min stirring and exchanging with ultrapure water every 6 h for 2 days. These samples after dialysis were used in SEM, TEM, DLS, UV-vis, Nanopore test, CD, and rheological tests. After dialysis, the samples are frozen at -80°C and then prepared as solid powder using a freeze-dryer under vacuum conditions. These solid powders were then used for XRD and XPS measurement. In details, TEM images are taken and recorded using the JEM-2100. AFM is performed by the NanoscopeⅧ microscopy system (MutiMode8, Bruker, USA) in tapping mode. Nanoscope analysis 1.8.0.0 was used for height analysis. The element distribution map was obtained by GeminiSEM 500 field emission scanning electron microscope. Dynamic light scattering (DLS, ZetasizerNano ZS, Malvern, UK) was used to determine the Zeta potential of different nanoparticles. UV-vis spectroscopy was used by the U-5100 UV-VIS spectrometer (Hitachi, Japan) to detect the binding between ceria nanozyme and amyloid fibrils, with a collection range of 200 nm to 700 nm. The nanopore tests were performed using solid-state nanopores. The secondary structure of amyloid fibrils was studied by circular dichroism (CD, ChirascanTM). In CD determination, a quartz cell with 1 mm path length was used to record CD signal values in the range of 190~260 nm at a scanning speed of 12 nm/min. The rheological properties of the hydrogel samples were tested using the MCR302 rheometer (Anton Paar, France). X-ray photoelectron spectroscopy (XPS) was obtained using the Thermo Scientific ESCALAB Xi instrument from the United States. All XPS spectra were calibrated using the C1s peak at 284.5 eV. Thermogravimetric analysis spectra are obtained using the DISCOVERY SDT 650 TA instrument (United States). These tests were conducted under a nitrogen gas atmosphere, with a heating rate of 10°C/min, and the temperature range was set from 30°C to 800°C. Confocal images were recorded by confocal laser scanning microscopy (NIS-Elements, Nikon, Japan).

*SOD-like activity and CAT-like activity tests:* SOD-like activity tests were performed *via* a superoxide dismutase (SOD) activity kit (Sigma-Aldrich, German). In brief, xanthine was firstly oxidized to uric acid and superoxide anion under the catalysis of xanthine oxidase (XOD). These produced superoxide anions were subsequently captured by the WST-1 that produced a water-soluble formazan dye upon reduction with superoxide anions. The rate of reduction with superoxide anion is linearly related to the XOD activity and is inhibited by SOD activity of samples. Therefore, the SOD activity of samples can be determined by a colorimetric method.

CAT-like activity tests were conducted according to the Amplex Red methods from previous work. In details, hydrogen peroxide (30 μL, 1 mM) and samples (100 μL) were respectively added in PBS solution (0.2 M, pH 7.4) and the final system volume was fixed at 3 mL, and incubated at 37 ℃ for 30 minutes. After the reaction, above solutions were centrifuged at 12000 rpm/min for 10 min to remove the samples. The upper supernatants were collected, and then 30 μL Amplex Red (1 mg/mL) and 30 μL horse radish peroxidase (HRP, 5 U/mL) were added into above supernatants and further incubated for 30 min. Finally, the reaction mixtures were centrifuged at 12000 rpm/min for 10 min to remove the HRP and the collected supernatants were measured using plated reader (BIO-TEK, ELX 800) at 571 nm absorbance.

*The determination of catalytic kinetics:* The steady-state kinetic assays were performed according to the previous study ^[1]^ with some revisions. The assays were conducted at 37℃ in PBS solution (0.2 M, pH7.4) with CeO_2_ or Lys-AFs-Ceria (2 mL) in various tubes. The steady-state kinetic analysis of CAT-mimetic activity was performed by changing the concentration of H_2_O_2_ (20, 50, 100, 200, and 400 mM) at a fixed concentration (final concentration of Ceria ions of both groups is 2 mM) of CeO_2_ or Lys-AFs-Ceria. The sample solution and different concentrations of H_2_O_2_ were added to the PBS solution so that the whole system was 10 mL, stirred in a water bath at 37°C, and the O_2_ concentration after different reaction times was determined using a dissolved oxygen meter, and the Michaelis-Menten constant was determined based on the Michaelis-Menten saturation curve. The related equations are as follow:

$$v_{0}=\frac{V_{max}[S]}{K_{m}+[S]} (1)$$

$\frac{1}{v_{0}}=\frac{K_{m}}{V_{max}}\cdot\frac{1}{\left[ S \right]}+\frac{1}{V_{max}}$ $(2)$

*V_max_* is the maximum reaction velocity. [*S*] is the substrate (H_2_O_2_) concentration. *V_0_* can represent the initial reaction velocity. *K_m_* is the Michaelis-Menten constant, representing the enzyme’s affinity for the substrate.

*In vitro cytocompatibility assays:* Mouse fibroblast L-929 cells, obtained from the Shanghai Stem Cell Bank of the Chinese Academy of Sciences, were used to test *in vitro* cytotoxicity of solution samples. L-929 cells (1x10^4^ cells/well) were cultured in 96-well plate using DMEM medium (Gibco, Invitrogen, USA) containing 10% FBS (Gibco, USA) and 1% penicillin/streptomycin at 37°C with 5% CO_2_. After incubation for 24 h, the original culture medium was removed, and 90 μL of fresh medium and 10 μL of the sample were added to each well. After further incubation at 37°C for 24 or 48 h, 10 μL MTT (5 mg/mL) was added to each well for 4 h at 37°C. The absorbance was measured at 490 nm wavelength using a microplate reader (BIO-TEK, ELX 800). In the cytotoxicity tests of the hydrogel samples, the hydrogel precursor solution (30 μL/well) was evenly coated on the bottom of a 96-well plate and allowed to form the hydrogel overnight under UV light. Next, 200 μL L-929 cells (1x10^4^ cells/well) were cultured on the surface of as-prepared hydrogel. The plate was maintained in a humidified environment at 37°C with 5% CO_2_ for 24 h or 48 h incubation. The original culture medium was removed, and 180 μL of fresh medium and 20 μL of MTT were added to each well. Following the 4-hour incubation at 37°C, the culture supernatant was collected by centrifugation and transferred to a new 96-well plate. The absorbance was then measured at 490 nm wavelength using a microplate reader (BIO-TEK, ELX 800). For CLSM images, calcein AM/PI was used to stain cells for 30 minutes and the fluorescence images of each group were observed by confocal laser scanning microscopy (NIS-Elements, Nikon, Japan).

*In vitro hemocompatibility assays:* The fresh mice blood was centrifuged at 4°C and 2500 rpm/min for 15 minutes, and the collected red blood cells were resuspended in 10 mL of PBS buffer. The centrifugation process was repeated five times until the supernatant was clear. Then, 900 μL of the red blood cell suspension and 100 μL of different samples were mixed and incubated for 4 hours at room temperature. Among them, PBS was set as the negative control and Triton X-100 was set as the positive control. Afterwards, these samples were centrifuged at 10,000 rpm/min for 10 minutes. The upper supernatant was collected and measured at 541 nm absorbance using a microplate reader (BIO-TEK, ELX800). The hemolysis percentage is calculated as the following formula:

$Hemolysis rate (\%)=\frac{Abs(Sample)-Abs(PBS)}{Abs (TritonX-100)-Abs(PBS)}$ $(3)$

*In vitro evaluation of ROS (H_2_O_2_) depletion and O_2_ production:* The detection of ROS was according to the previous study ^[2]^. The different hydrogel samples were divided into a 96 well plate (30 μL/well) and sterilized overnight with ultraviolet radiation. L-929 (1×10^4^ cells/well) were inoculated on the surface of hydrogel in 96-well plate and incubated overnight. Then, H_2_O_2_ solution was added in every group, and the final H_2_O_2_concentration was set as 100 µM. Among them, PBS group with and without hydrogen peroxide were set as the positive and negative control group, respectively. After 12 h incubation, cells were then incubated with DCFH-DA (10 µM in FBS-free DMEM) for 20 minutes and washed twice using PBS. The intracellular ROS level was assessed via the fluorescence detection of DCF (λex=488 nm, λem=525 nm) by confocal laser scanning microscopy (NIS-Elements, Nikon, Japan). Similar with above method, after further cultivation for 12 hours, cells were incubated with [Ru(dpp)_3_]Cl_2_ (10 µM in FBS-free DMEM) for 20 minutes and washed twice using PBS. Intracellular O_2_ evaluation (λex=450 nm, λem=610 nm) was also evaluated by the confocal laser scanning microscopy (NIS-Elements, Nikon, Japan).

*Macrophage polarization:* The hydrogel was prefabricated on a 6-well plate with the amount of 1 mL/well and sterilized by ultraviolet light overnight. Macrophage RAW264.7 cells were cultured on the surface of hydrogel with the density of 1×10^5^/well and incubated for 48 hours. Next, RAW264.7 cells were collected from the hydrogel with precooled PBS buffer, and re-suspended in 500 μL PBS. Then, CD206 antibody and CD86 antibody were added in above cells and incubated on ice in dark for 30 min. Flow cytometry (FCM) and confocal laser scanning microscopy (CLSM) were used to measure and observe the expression of CD206 and CD86 in RAW264.7 cells, respectively.

*In vitro tube formation assays:* The *in vitro* tube formation assays were performed according to previous studies ^[3]^. Matrigel matrix (BD Biosciences, USA) was thawed at 4°C overnight and then mixed with different proportions of hydrogels. 10 µL mixed Matrigel™ Matrix was added to each well of µ-Slide Angiogenesis (ibidi, Germany) and incubated at 37°C for 1 h. Subsequently, HUVEC cells (1×10^4^) were added to each well and cultured for 8 h at 37°C in a 5% CO_2_ humidified incubator. After cultured for 8 h, the cells were stained with TRITC Phalloidin and DAPI, and observed and photographed using fluorescence microscope. Total tubular length and branch points were analyzed by Image J software.

*In vitro antibacterial activity measurements:* Gram-positive bacteria *Staphylococcus aureus* and Gram-negative bacteria *Escherichia coli* were used to evaluate the *in vitro* antibacterial efficiency of the hydrogel. Prior to each experiment, the bacteria were cultured overnight on LB solid medium plates at 37℃. A single colony was picked and incubated overnight in 4 mL of LB medium at 37℃. After washing twice with sterile PBS (10 mM, pH 7.4), the bacteria were diluted to a concentration of 10^7^ CFU/mL (OD_600_≈0.1) for the following experiments. The hydrogel was prepared according to the above preparation method and pre-sterilized overnight using ultraviolet light on a 24-well plate. Each well was then inoculated with 500 µL of the prepared bacterial suspension (1×10^7^ CFU/mL) and incubated at 37℃ for 8 h. Subsequently, the antibacterial efficiency was evaluated using the spread plate method (SPM). Also, the bacteria were washed with PBS twice (8000 rpm/min, 5 min) and fixed overnight in 2.5% glutaraldehyde at 4℃. On the next day, the bacteria were dehydrated in a gradient of ethanol (50, 60, 70, 80, 90, and 100 v/v %) for SEM measurements.

*CFU counting:* The CFU counting was performed according to the previous work ^[4]^. Prior to each experiment, the bacteria were grown overnight at 37°C on LB plates. Single bacterial colony of each strain were collected and incubated overnight in 4 mL LB at 37 °C. After being washed with sterile PBS (10 mM, pH 7.4) twice, the bacteria were diluted to a concentration of 10^7^ colony forming units (CFUs) mL^−1^ for the following experiments. The hydrogel was preformed in 24-well plate overnight and disinfected for 2 h by UV light. And the peptide monomers were dissolved by a certain amount of DMSO and diluted into coreesponding concentrations using ultrapure water. Above 500 µL prepared bacteria suspension (1×10^7^ CFUs·mL^−1^) was added to each well and incubated for different times (1, 3, 6, and 8 h) at 37°C. Then, the spread plate method (SPM) was used to estimate the antibacterial effect of each sample.

*Live/dead bacterial staining analysis:* The hydrogel was formed in 24-well plate overnight and disinfected for 2 h using UV light. 500 µL prepared bacteria suspension (1×10^7^ CFUs·mL^−1^) was added to each well and incubated 6 h at 37°C. According to the illustration of the Live/Dead BacLight bacteria viability kits (L13152, Invitrogen), the bacteria suspensions after hydrogel treatments were gently rinsed three times with PBS, and then stained with 500 µL staining reagent mixtures for 30 min in darkness. Finally, the bacteria suspension was observed by fluorescence microscope (Leica DMI8, Germany). The green fluorescent represented the live bacteria, whereas the red fluorescent represented the dead bacteria.


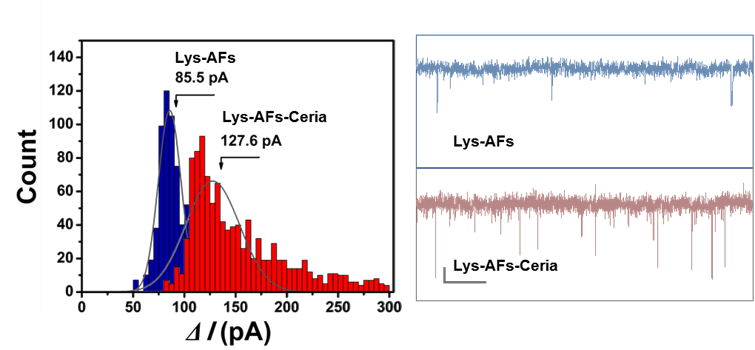


**Fig S1.** Nanopore tests of Lys-AFs and Lys-AFs-Ceria.


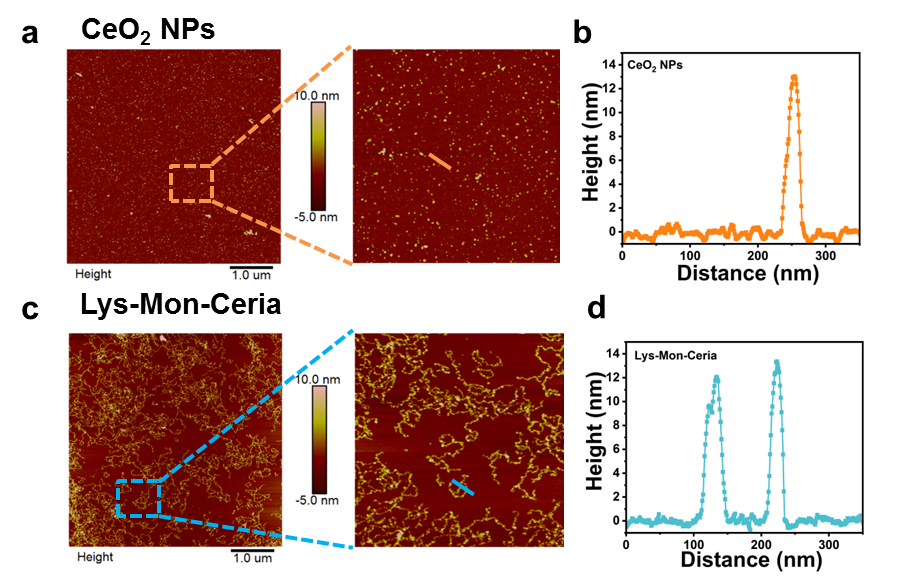


**Fig S2**. AFM images of CeO_2_ NPs and Lys-Mon-Ceria, and corresponding height analysis.


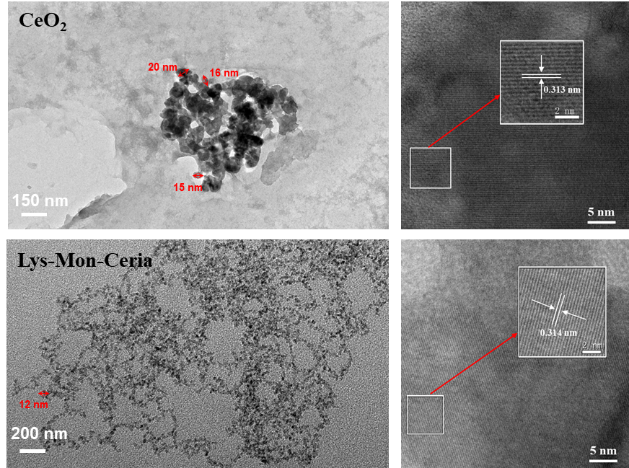


**Fig S3**. TEM images of CeO_2_ NPs and Lys-Mon-Ceria, and corresponding crystal structure analysis.


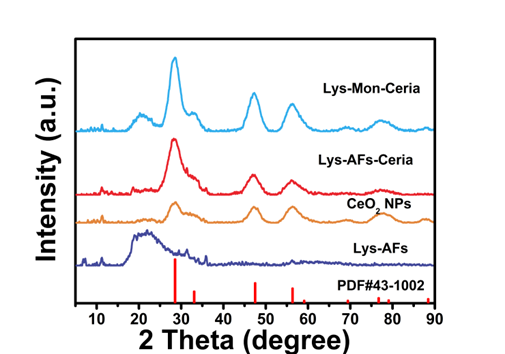


**Fig S4.** Powder XRD analysis of different groups including Lys-AFs, CeO_2_ NPs, Lys-AFs-Ceria, and Lys-Mon-Ceria.

**
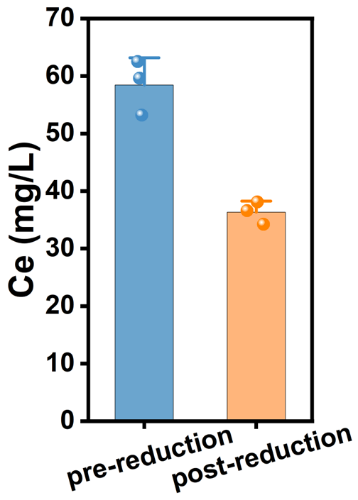
**

**Fig S5.** ICP-MS analysis of Ce ions content in the Lys-AFs-Ceria solution of pre-reduction and post-reduction.


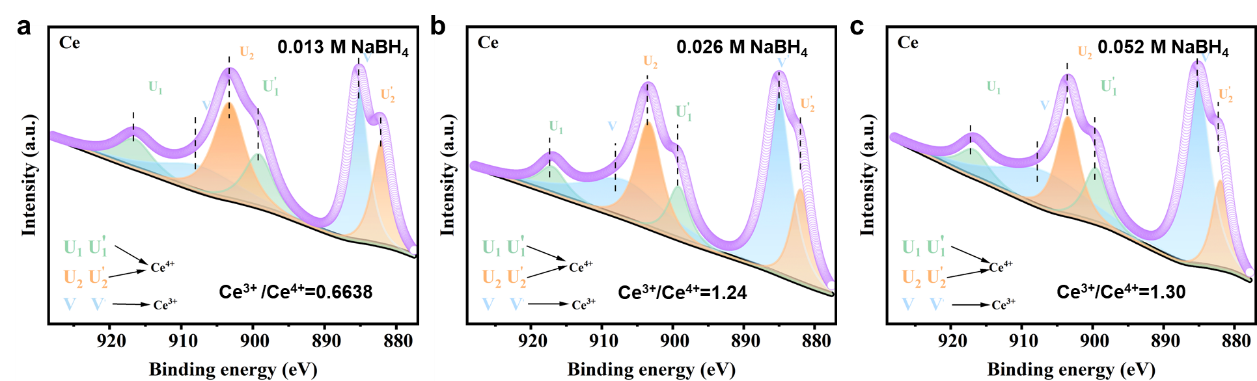


**Fig S6.** XPS spectrum of Lys-AFs-Ceria after adding various concetrations of NaBH_4_.


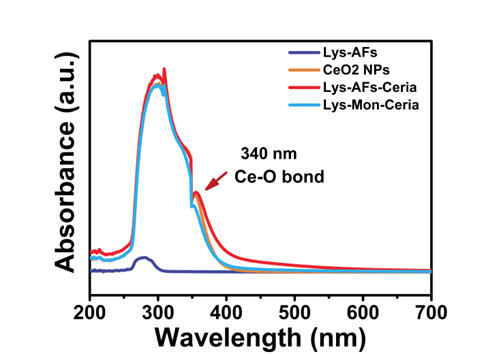


**Fig S7.** UV-vis spectra of different groups including Lys-AFs, CeO_2_ NPs, Lys-AFs-Ceria, and Lys-Mon-Ceria.

**
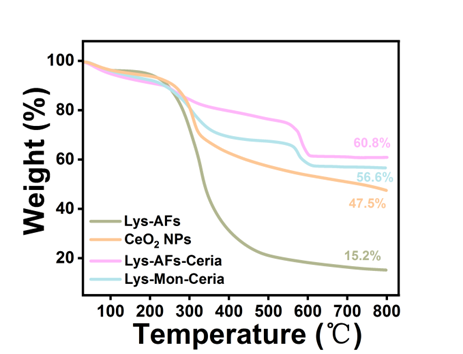
**

**Fig S8.** TGA analysis of different groups including Lys-AFs, CeO_2_ NPs, Lys-AFs-Ceria, and Lys-Mon-Ceria.


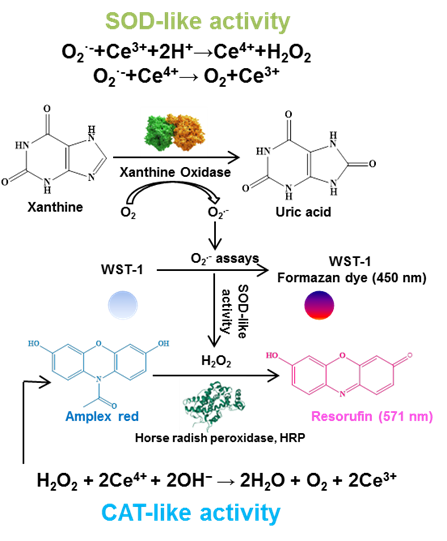


**Fig S9.** Schematic representation of the O_2_^.-^ and H_2_O_2_ assays.

**
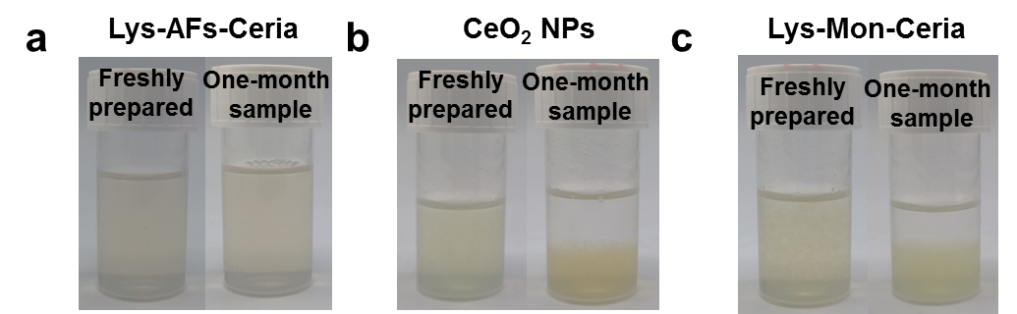
**

**Fig S10.** Representative images of freshly-prepared and one-month samples of CeO_2_ NPs, Lys-AFs-Ceria, and Lys-Mon-Ceria.


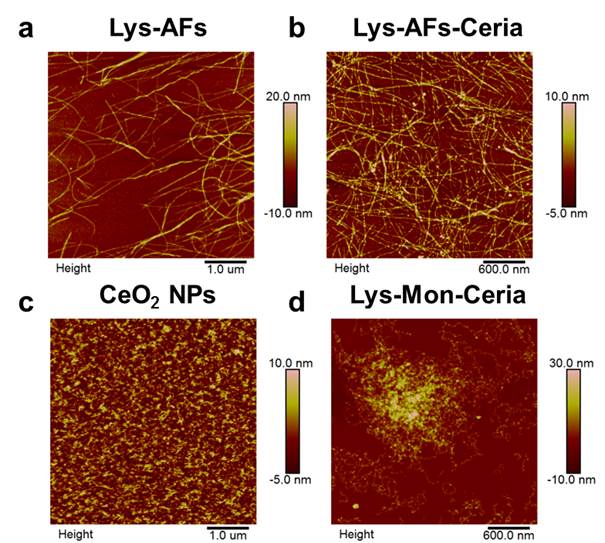


**Fig S11.** AFM images of one-month samples of Lys-AFs, CeO_2_ NPs, Lys-AFs-Ceria, and Lys-Mon-Ceria.


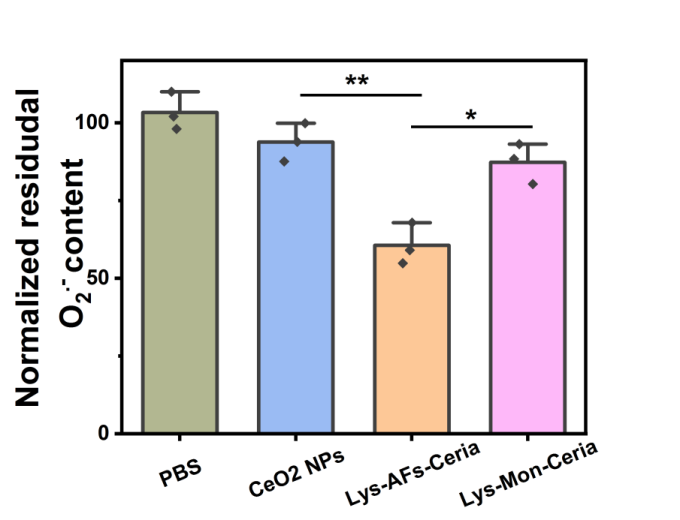


**Fig S12.** O_2_^.-^ elimination capacity assays of different samples kept for one month.


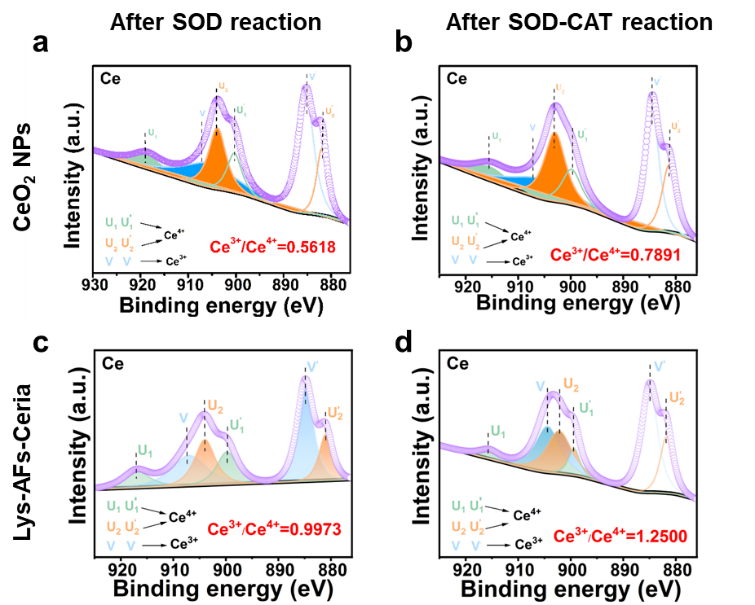


**Fig S13.** XPS spectrum of CeO_2_ NPs and Lys-AFs-Ceria after SOD and SOD-CAT activity assays.


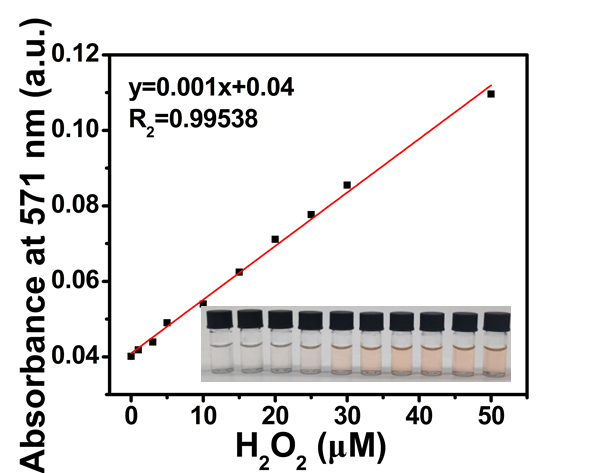


**Fig S14**. Standard curve of resorufin versus H_2_O_2_ concentrations detected at 571 nm.


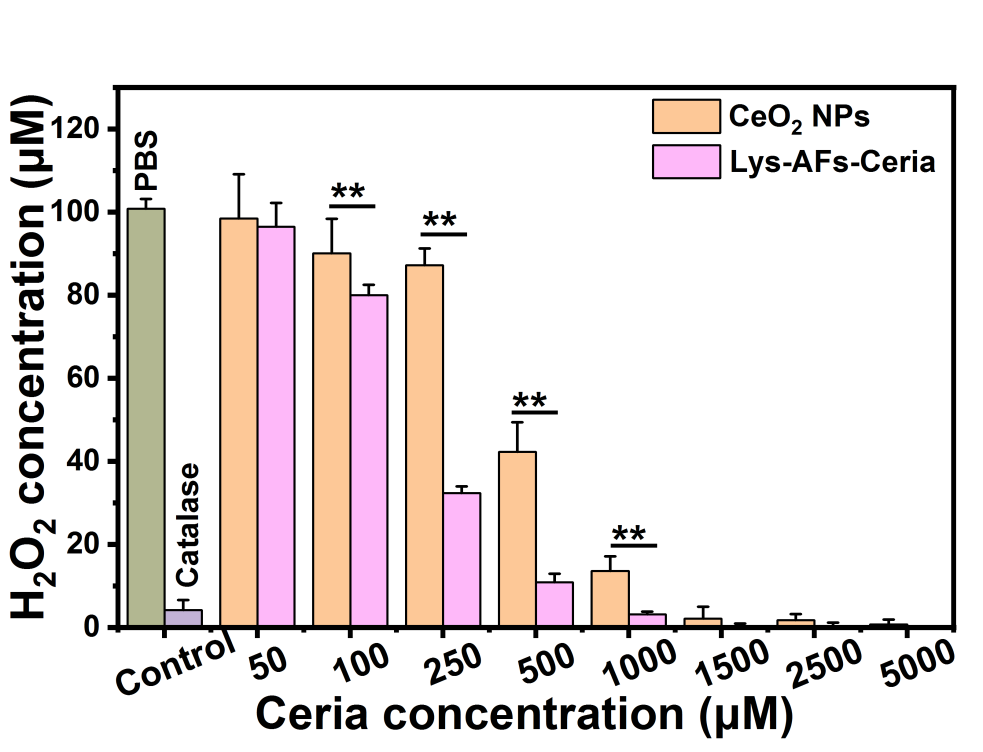


**Fig S15.** Residual H_2_O_2_ production assays (n=4) of different ceria concentrations in CeO_2_ NPs and Lys-AFs-Ceria group. Note: all samples used in CAT assays were the materials after SOD-activity assays.


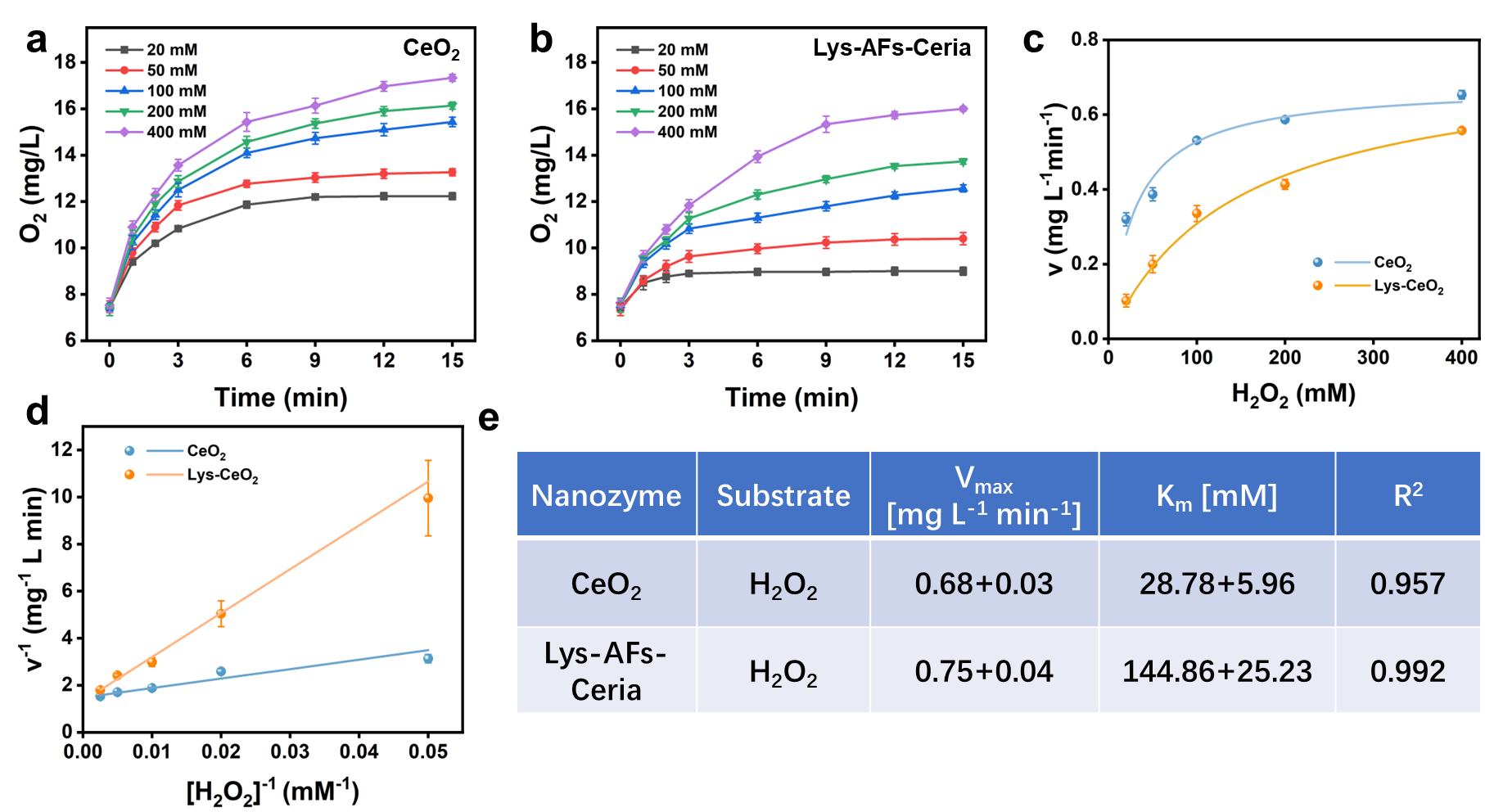


**Fig S16.** O_2_ generation with different treatments of CeO_2_ (a) and Lys-AFs-Ceria (b) under the different concentrations of H_2_O_2_ (20, 50, 100, 200, and 400 mM). (c) Michaelis–Menten kinetic analysis and (d) Lineweaver–Burk plotting of CAT-like activity with H_2_O_2_ as a substrate. (e) Comparison of the kinetic parameters of CAT-like activity.


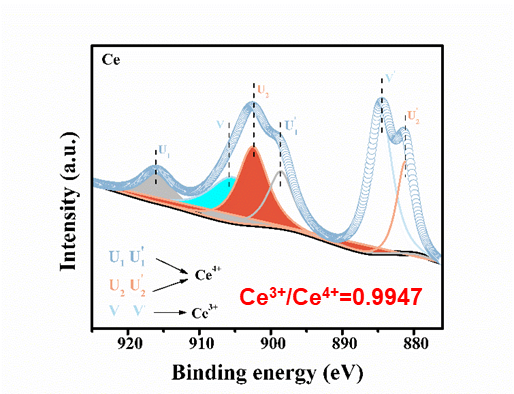


**Fig S17.** XPS results of Lys-AFs-Ceria after five cycles of SOD-CAT catalytic process.


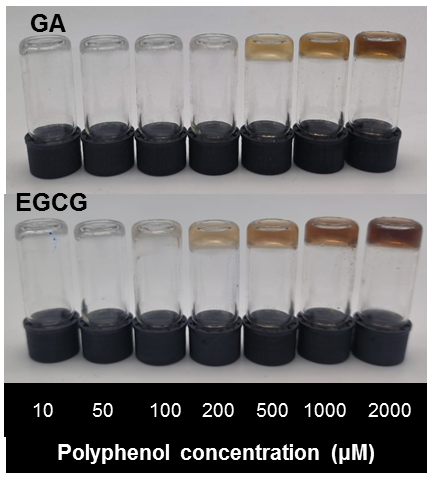


**Fig S18.** Vial inversion images of hydrogel induced by different kinds of polyphenol, including GA and EGCG.


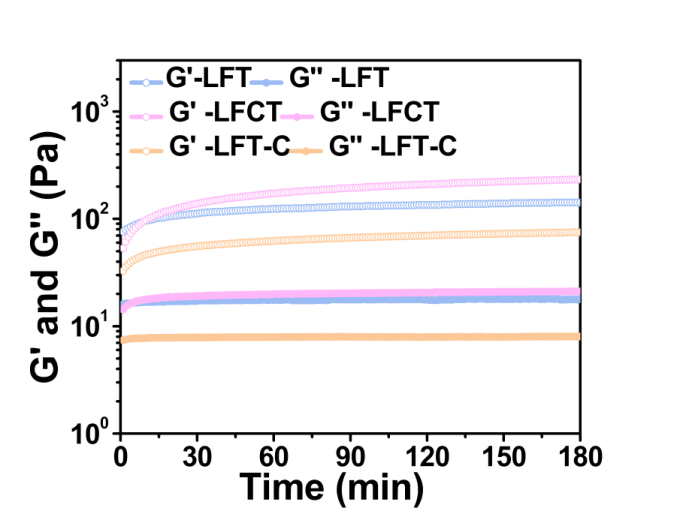


**Fig S19.** Time-scan rheological experiments of LFT, LFT-CeO_2_ NPs, and LFCT hydrogel.


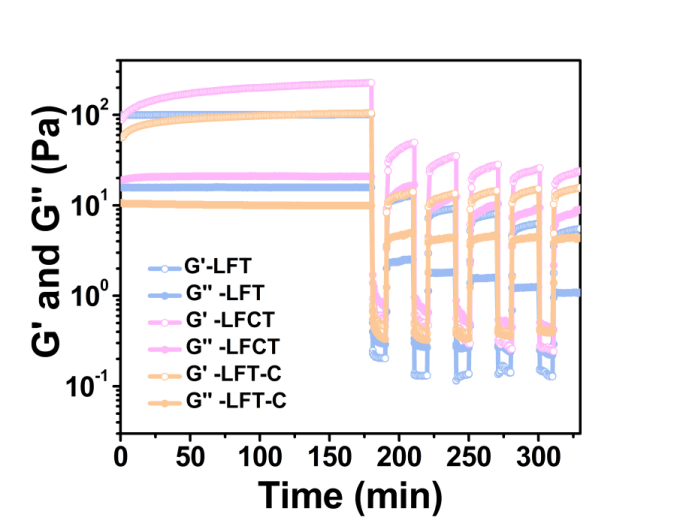


**Fig S20.** The mechanical property of different kinds of hydrogel was evaluated by testing the G′ and G″ cycling during five times of shear strain between 1% and 100% at 25℃.


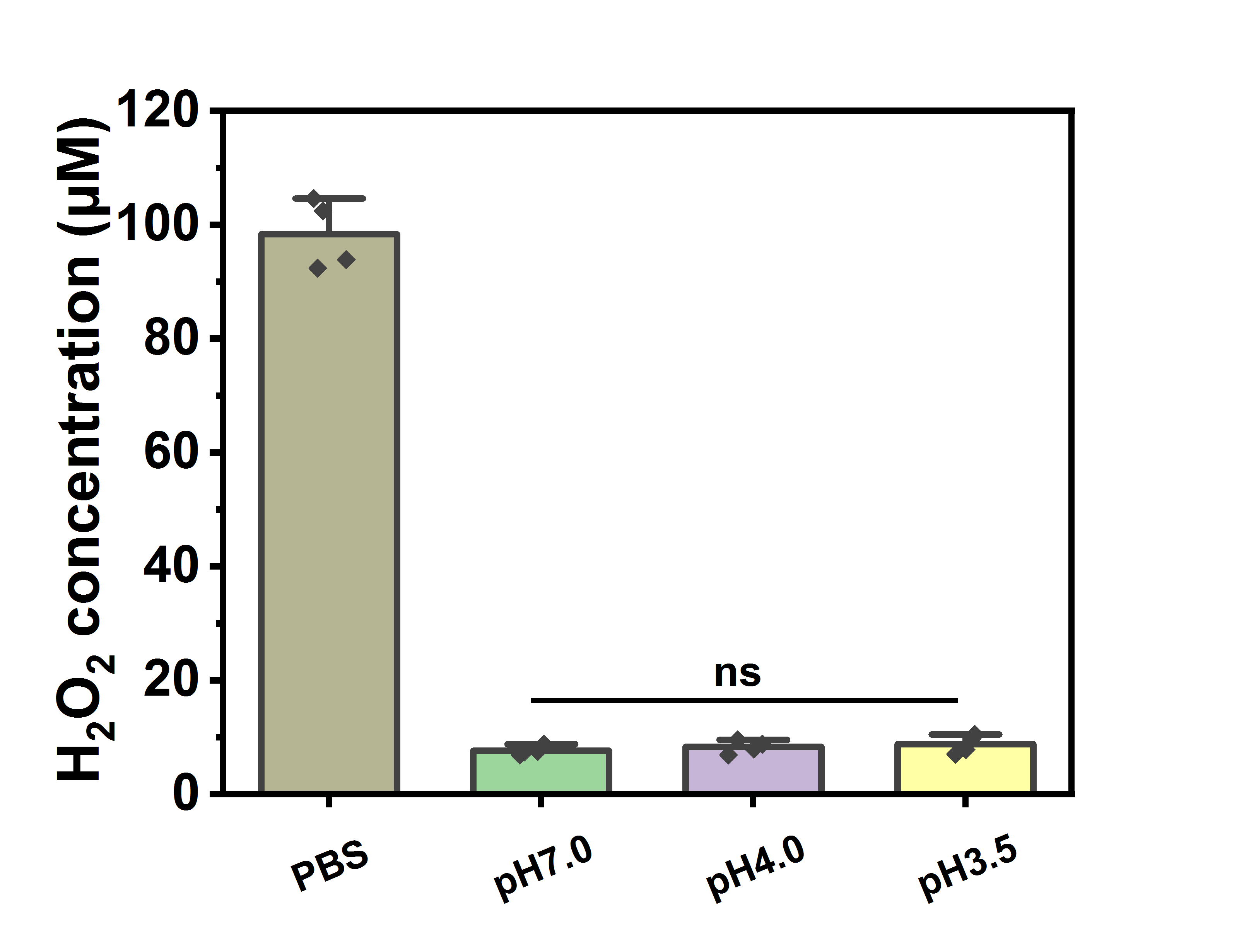


**Fig S21.** The CAT-mimetic activity assays of LFCT hydrogel in various pH conditions (pH=3.5, 4, and 7).


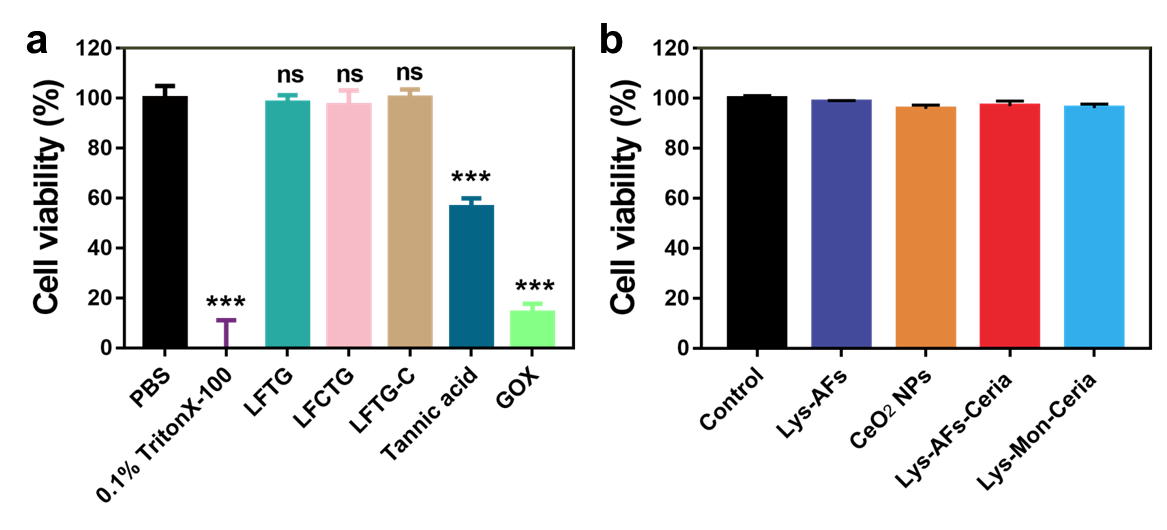


**Fig S22.** Cell viability of L-929 cells on different hydrogels and related components using MTT Method.


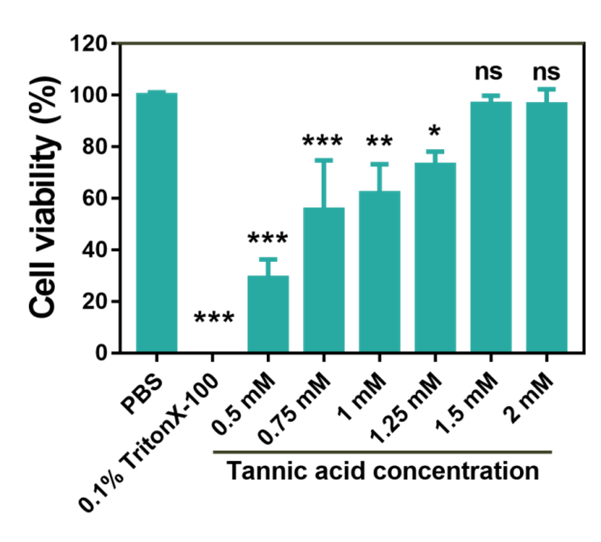


**Fig S23.** Cell viability of L-929 cells on LFCTG hydrogels with different TA concentrations using MTT Method.


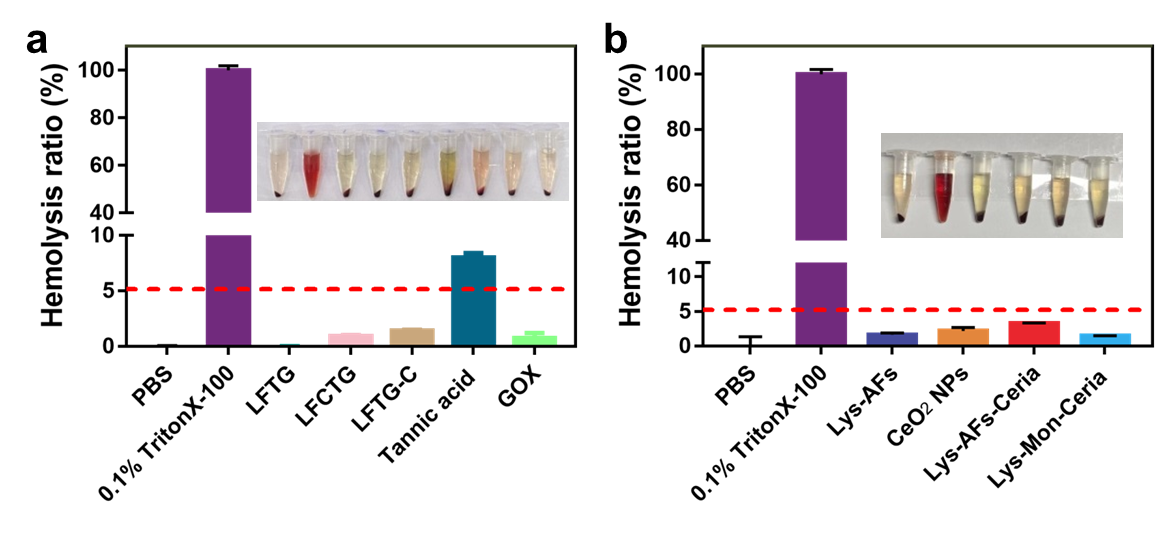


**Fig S24.** Hemolysis ratio of red blood cells on different hydrogels and related components.


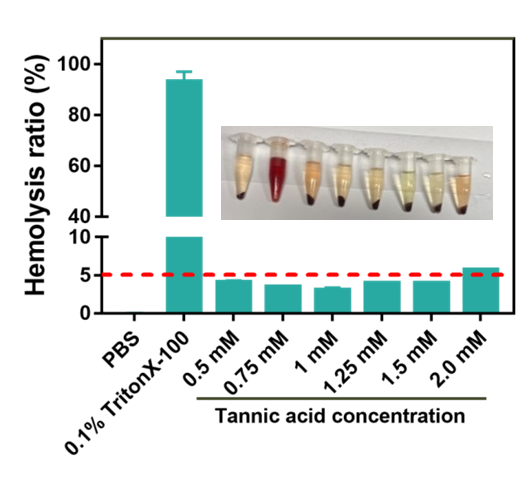


**Fig S25.** Hemolysis ratio of red blood cells on LFCTG hydrogels with different TA concentrations.


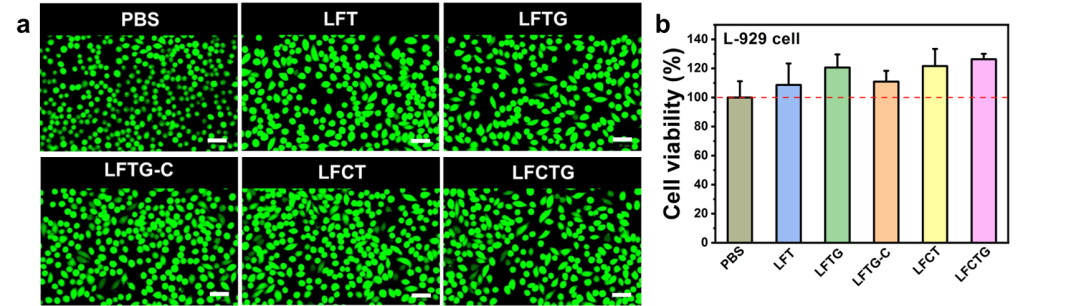
**Fig S26.** Confocal images of L-929 cells after the incubated with different hydrogels for 12 h and quantitative cell viability analysis for 48 h.


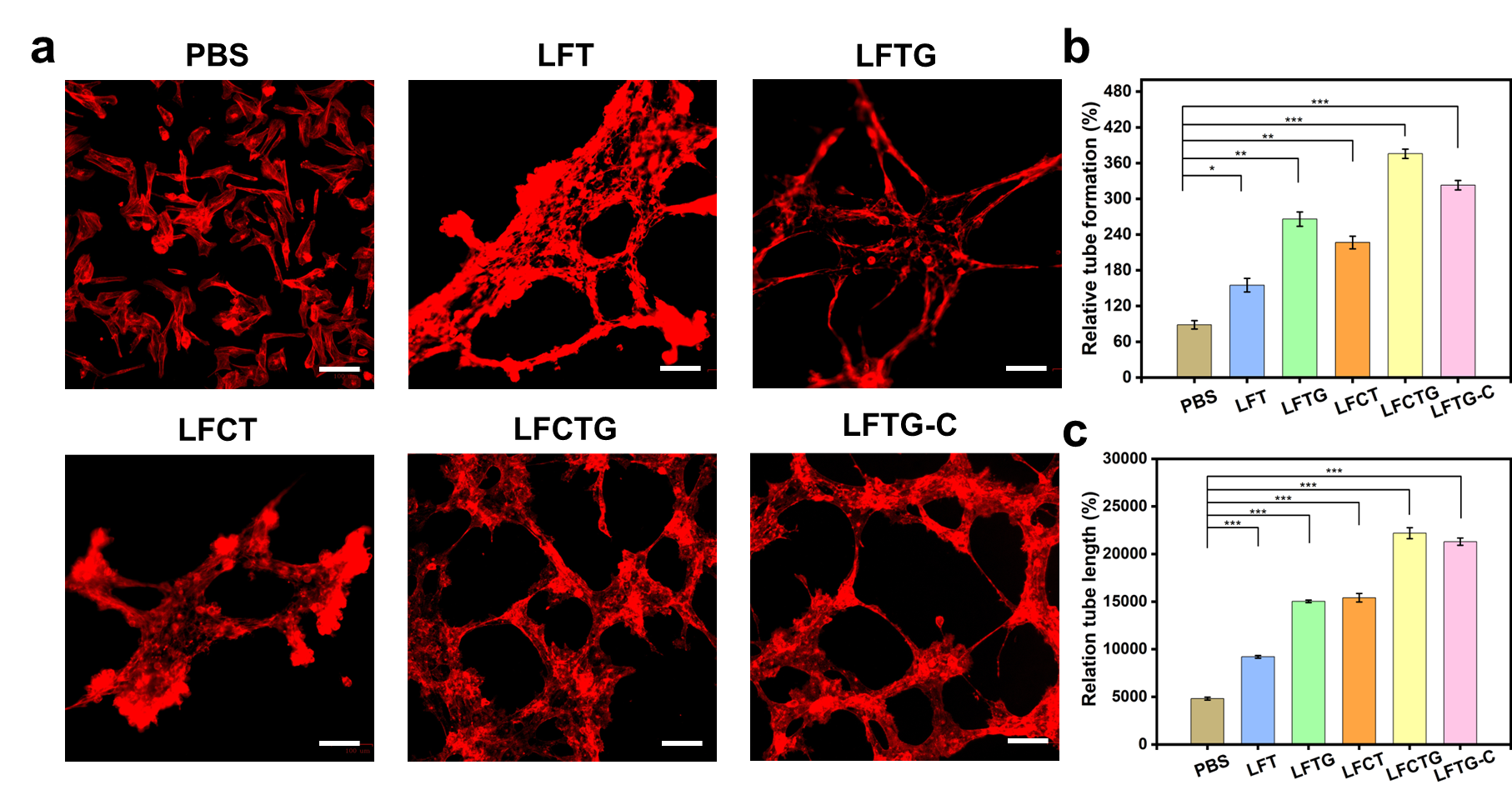


**Fig S27.** (a) The tube formation pictures (Scale: 100 µm), (b) tube formation number, and (c) tube formation length of of HUVEC cells after incubation with different kinds of hydrogel.


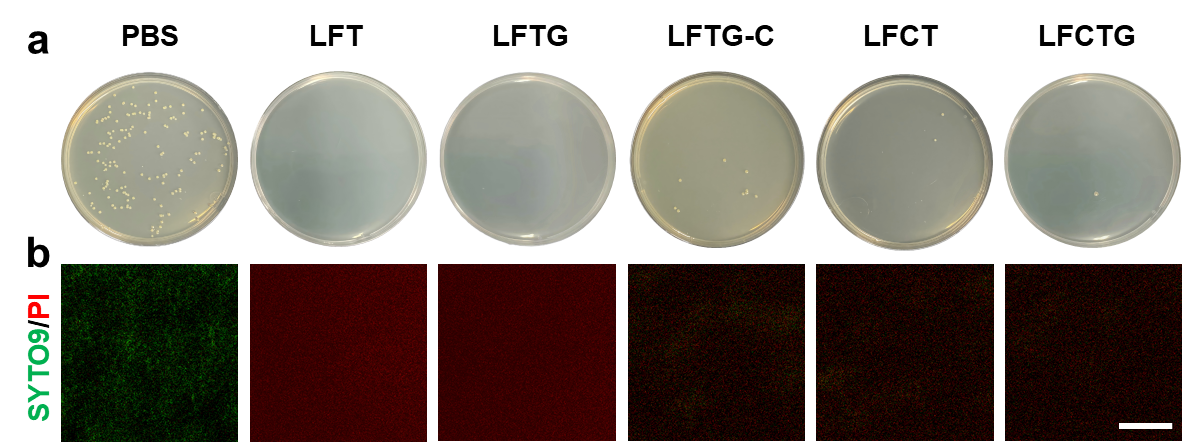


**Fig S28.** (a) Agar plates pictures, and (b) live-dead staining results of MRSA treated with different concentrations of hydrogel. (Scale: 50 µm.)


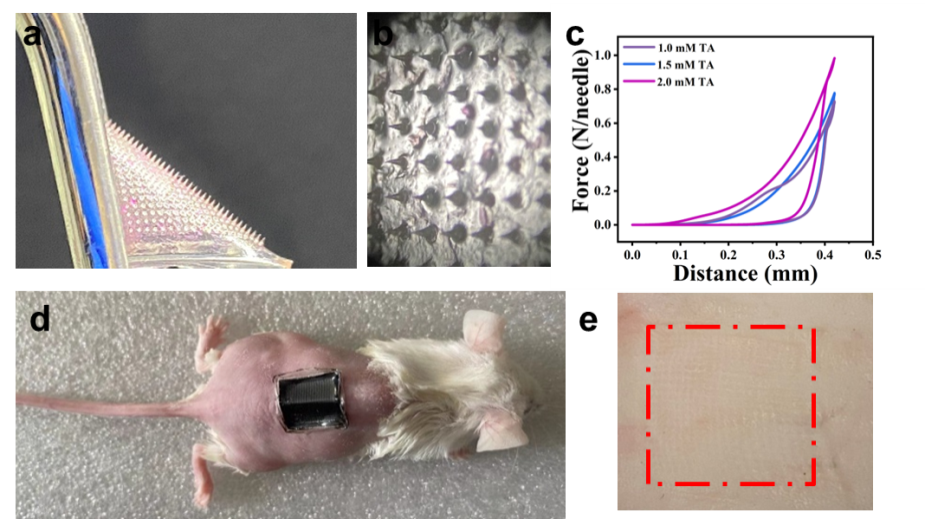


**Fig S29.** (a) The image of microneedle patch. (b) Microscopic images of microneedle tips. (c) Mechanical compression force curves of LFCTG hydrogel with different concentrations of tannic acid. (d) Illustration of microneedle applied on the skin of mouse. (e) Skin images of mouse after applied microneedle patch on the skin for 2 h.


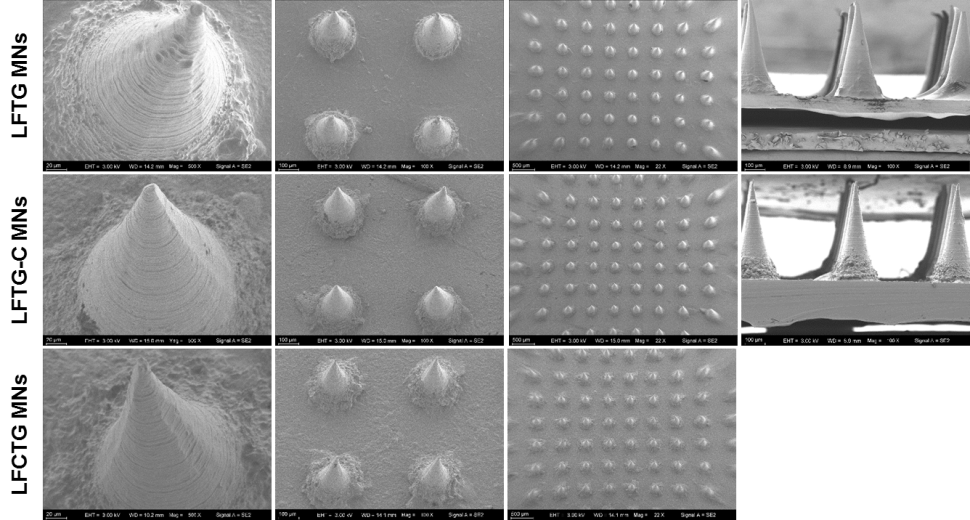


**Fig S30.** SEM images of microneedles (MNs) fabricated by LFTG and LFTG-CeO_2_ NPs.


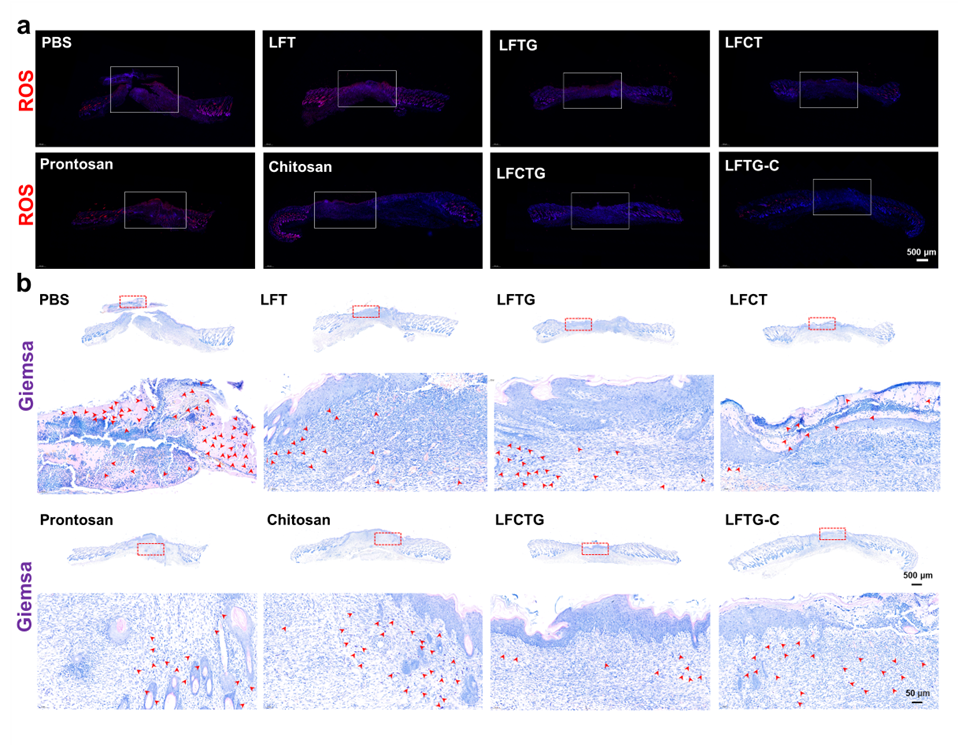


**Fig S31.** (a) DHE (a ROS probe) immunofluorescence images (Scale: 500 µm) and (b) Giemsa staining images of the skin tissues from MRSA-infected diabetic mice wounds in different treatment groups. (Scale: 500 µm and 50 µm)


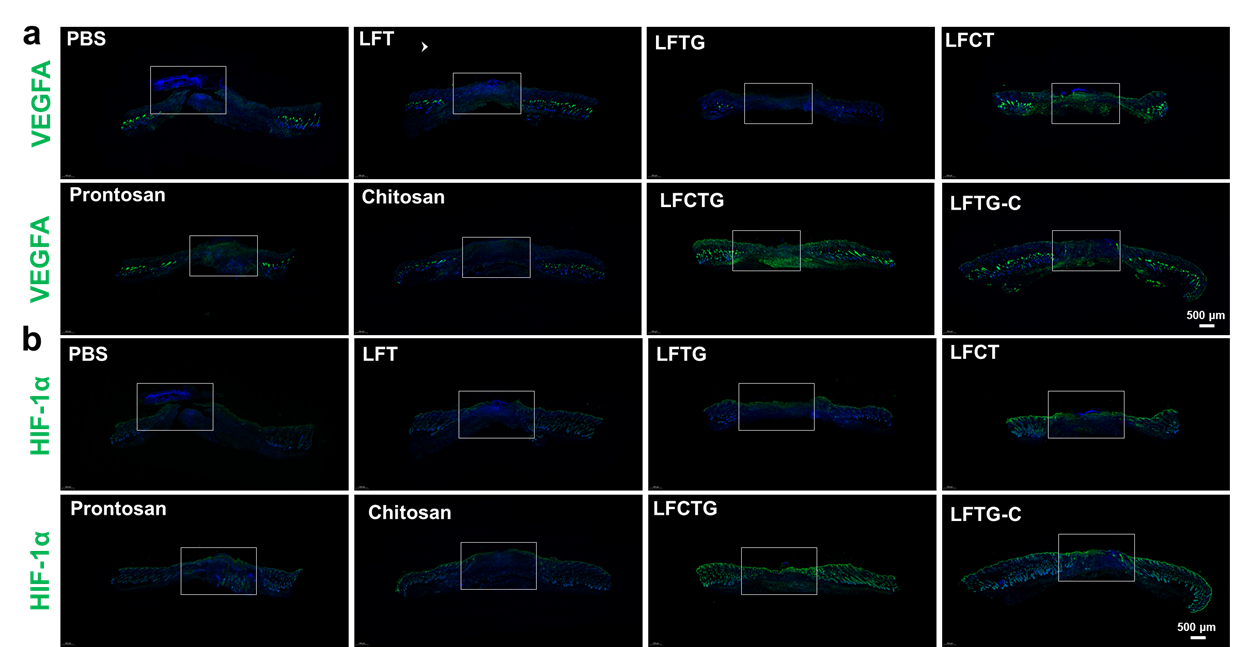


**Fig S32.** (a) VEGFA (green) and (b) HIF-1α (green) immunofluorescent staining of the wound tissues in representative treatment groups. (Scale: 500 µm.)


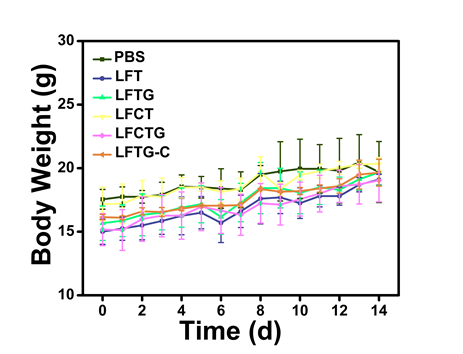


**Fig S33.** The body weight changes of diabetic mice during 14 days after various microneedles treatments including PBS, LFT, LFTG, LFCT, LFCTG, and LFTG-CeO_2_ NPs.


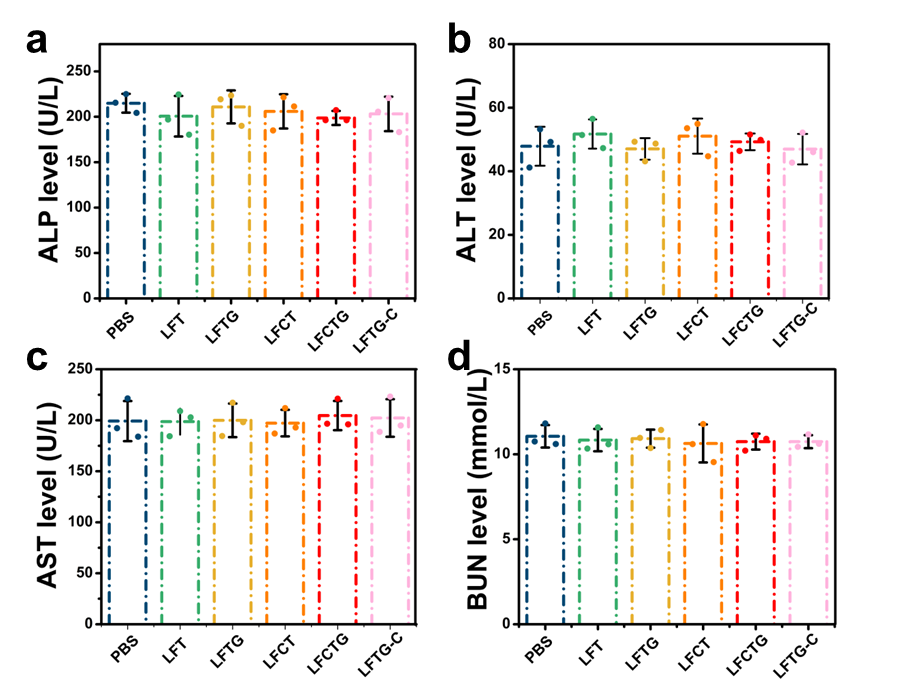


**Fig S34.** Blood biochemistry analysis including (a) AST, (b) ALT, (c) ALP, and (d) BUN of diabetic mice on day 14 after the various microneedle treatments including PBS, LFT, LFTG, LFCT, LFCTG, and LFTG-CeO_2_ NPs.


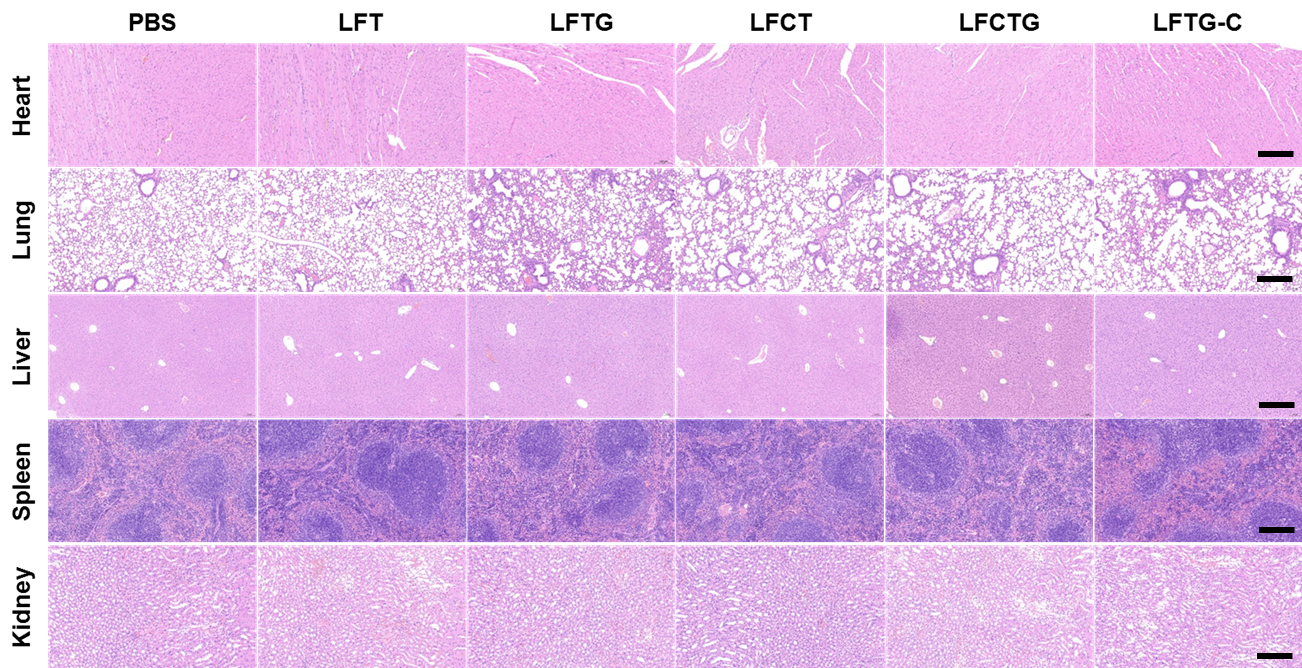


**Fig S35.** H&E staining slices of heart, liver, spleen, lung, and kidney of diabetic mice on day 14 after the various microneedle treatments including PBS, LFT, LFTG, LFCT, LFCTG, and LFTG-CeO_2_ NPs (Scale bar: 200 µm).

**References**

[1] S. Dong, Y. Dong, B. Liu, J. Liu, S. Liu, Z. Zhao, W. Li, B. Tian, R. Zhao, F. He, S. Gai, Y. Xie, P. Yang, Y. Zhao, *Adv. Mater* **2022**, 34, e2107054.

[2] T. Ma, X. Zhai, M. Jin, Y. Huang, M. Zhang, H. Pan, X. Zhao, Y. Du, *VIEW* **2022**, 3, 20220045.

[3] a)Y. Yi, Z. Yang, C. Zhou, Y. Yang, Y. Wu, Q. Zhang, *Nano TransMed* **2024**, 3, 100030; b)X. Wu, D. Huang, Y. Xu, G. Chen, Y. Zhao, *Adv. Mater* **2023**, 35, 2301064; c)Q. Xuan, F. Jiang, H. Dong, W. Zhang, F. Zhang, T. Ma, J. Zhuang, J. Yu, Y. Wang, H. Shen, C. Chen, P. Wang, *Adv. Funct. Mater.* **2021**, 31, 2106705.

[4] Y. Sang, W. Li, H. Liu, L. Zhang, H. Wang, Z. Liu, J. Ren, X. Qu, *Adv. Funct. Mater.* **2019**, 29, 1900518.
